# Baseline human gut microbiota profile in healthy people and standard reporting template

**DOI:** 10.1371/journal.pone.0206484

**Published:** 2019-09-11

**Authors:** Charles H. King, Hiral Desai, Allison C. Sylvetsky, Jonathan LoTempio, Shant Ayanyan, Jill Carrie, Keith A. Crandall, Brian C. Fochtman, Lusine Gasparyan, Naila Gulzar, Paul Howell, Najy Issa, Konstantinos Krampis, Lopa Mishra, Hiroki Morizono, Joseph R. Pisegna, Shuyun Rao, Yao Ren, Vahan Simonyan, Krista Smith, Sharanjit VedBrat, Michael D. Yao, Raja Mazumder

**Affiliations:** 1 The Department of Biochemistry & Molecular Medicine, School of Medicine and Health Sciences, George Washington University Medical Center, Washington, DC, United States of America; 2 McCormick Genomic and Proteomic Center, George Washington University, Washington, DC, United States of America; 3 The Department of Exercise and Nutrition Sciences, Milken Institute School of Public Health, George Washington University, Washington, DC, United States of America; 4 The Institute for Biomedical Science, School of Medicine and Health Sciences, George Washington University, Washington, DC, United States of America; 5 Center for Genetic Medicine, Children’s National Medical Center, George Washington University, Washington, DC, United States of America; 6 Computational Biology Institute and The Department of Biostatistics and Bioinformatics, Milken Institute School of Public Health, George Washington University, Washington, DC, United States of America; 7 KamTek Inc, Frederick, Maryland, United States of America; 8 Department of Biological Sciences, Hunter College, City University of New York, New York, New York, United States of America; 9 Center for Translational Medicine, Department of Surgery, George Washington University, Washington, DC, United States of America; 10 Division of Gastroenterology and Hepatology VA Greater Los Angeles Healthcare System and Department of Medicine and Human Genetics, University of California, Los Angeles, Los Angeles, California, United States of America; 11 Washington DC VA Medical Center, Gastroenterology & Hepatology Section, Washington, DC, United States of America; 12 Department of Medicine, School of Medicine and Health Sciences, George Washington University, Washington, DC, United States of America; Beckman Research Institute, UNITED STATES

## Abstract

A comprehensive knowledge of the types and ratios of microbes that inhabit the healthy human gut is necessary before any kind of pre-clinical or clinical study can be performed that attempts to alter the microbiome to treat a condition or improve therapy outcome. To address this need we present an innovative scalable comprehensive analysis workflow, a healthy human reference microbiome list and abundance profile (GutFeelingKB), and a novel Fecal Biome Population Report (FecalBiome) with clinical applicability. GutFeelingKB provides a list of 157 organisms (8 phyla, 18 classes, 23 orders, 38 families, 59 genera and 109 species) that forms the baseline biome and therefore can be used as healthy controls for studies related to dysbiosis. This list can be expanded to 863 organisms if closely related proteomes are considered. The incorporation of microbiome science into routine clinical practice necessitates a standard report for comparison of an individual’s microbiome to the growing knowledgebase of “normal” microbiome data. The FecalBiome and the underlying technology of GutFeelingKB address this need. The knowledgebase can be useful to regulatory agencies for the assessment of fecal transplant and other microbiome products, as it contains a list of organisms from healthy individuals. In addition to the list of organisms and their abundances, this study also generated a collection of assembled contiguous sequences (contigs) of metagenomics dark matter. In this study, metagenomic dark matter represents sequences that cannot be mapped to any known sequence but can be assembled into contigs of 10,000 nucleotides or higher. These sequences can be used to create primers to study potential novel organisms. All data is freely available from https://hive.biochemistry.gwu.edu/gfkb and NCBI’s Short Read Archive.

## Introduction

While humanity has only begun to influence planetary-level events in the last few hundred years [1], microorganisms have shaped our planet since time immemorial [2]. It has been shown that the microbes of the ocean are as important for influencing planetary climate as the microbes of gastrointestinal (GI) tracts of cattle [3]; furthermore, new functions are continuously found for the human microbiome [4–6]. However, since the advent of germ theory and the antimicrobial revolution, microbes have been viewed as insurgents bound for eradication [7]. Hence, we have created GutFeelingKB to provide a reference for the metagenomic analysis of the human gut microbiome.

In 2001, some sixty years into the antibiotic era, Joshua Lederberg coined the term ‘microbiome’ as the pendulum of opinion began to swing back to a more microbe-tolerant position [8,9]. In 2008, the US National Institutes of Health launched the Human Microbiome Project (HMP) to better understand the makeup of the community of microbes in cohabitation with humans [10,11]. This population of microorganisms brings with it a vast, diverse, and modifiable set of genomes which have proven to influence human health and disease [12,13]. Together, these organisms’ genomes comprise the metagenome, a highly versatile pool of genetic elements which now serves as a target for medical research [14]. Microbiome characterization through various analysis pipelines has advanced progressively since HMP and this development process has catalyzed the understanding of certain roles of these microbial communities [15,16].

Although microbiomes of all body sites are important, the gut microbiome, with hundreds of prevalent species is of major interest to a large and diverse number of researchers [17,18]. The healthy gut microbiome data and analysis is crucial for all studies of disease with relation to the human gut. A *Nature Microbiology* issue in 2016 contained a consensus statement which outlined all federally-funded microbiome research over a three-year period [19]. The authors, on behalf of the federal government’s FastTrack Action Committee on Mapping Microbiomes (FTAC-MM), defined a microbiome as a multi-species community of microorganisms in any environment: host, habitat, or ecosystem. One of the conclusions reached by the authors was a “priority need” for higher-throughput, more accurate data acquisition, better pipelines for data analyses, and a greater ability to organize, store, access, and share/integrate data sets. At present, most studies leverage study specific control groups and reporting mechanisms. The studies that are successful at creating clinically relevant results, such as the work by uBiome [20], are based on marker genes, and so they do not shed light on the origin of the “microbial dark matter”, and are not able to be integrated with whole genome shotgun sequencing studies (WGS). These problems are compounded by the fact that different bioinformatics pipelines produce different results largely because all current pipelines use a limited number of *ad hoc* reference organisms to determine abundance. It has also been shown that database growth influences the accuracy of relatively faster k-mer-based species identification [21]. The final understanding of the baseline healthy microbiome therefore can be flawed because the methods are uniquely applied in each study. As such, there is a need for aggregation, validation for interoperability, and eventual standardization of methods and reporting.

Currently, metagenomic analyses use nucleotide sequences from a limited set of pre-determined microorganisms or genes as a reference database, and, as such, these reference lists are not truly comprehensive. The use of limited sets of sequence data is prevalent because it is computationally challenging to perform pairwise read alignment against the entire NCBI non-redundant nucleotide database (NCBI-nt) [22]. Algorithms have been developed that allow the use of the complete NCBI-nt and it has been shown that using the NCBI-nt permits accurate analysis of the data with significantly fewer errors in microorganism abundance quantification [23]. To leverage this prior work on metagenomic analysis algorithms, samples from a healthy cohort of participants were collected and sequenced to specifically target healthy control data. To ensure the samples were abundant and correct enough to build healthy reference list, we also retrieved sequences of healthy people from HMP. Furthermore, we developed an approach that generates a collection of assembled contiguous sequences (contigs) that cannot be aligned to any known sequence in NCBI-nt but are present in healthy individual fecal samples and are ideal for healthy-disease-microbiome correlation analysis and novel primer design. For the purposes of this study, these sequences are defined as metagenomic dark matter–sequences that cannot be mapped to any known sequence but can be assembled into contigs of 10,000 nucleotides or higher. Together, these data form our Gut Feeling Knowledge Base–GutFeelingKB. The contig nucleotide length threshold is expected to reduce the number of contigs in GutFeelingKB that are not of biological origin. Our definition is much stricter than previous definitions of the metagenomic dark matter which accepts remote homology to known sequences [24]. The need to include metagenomic dark matter in comprehensive analyses of the gut microbiome matches the arguments presented by Bernard et al. in their recent manuscript on microbial dark matter where they opine that “unraveling the microbial dark matter should be identified as a central priority for biologists” [25].

The primary aim in creating GutFeelingKB is to provide a reference knowledgebase for the metagenomic analysis of the human gut microbiome. All the organisms which were confidently observed in a healthy human gut are included. Using this knowledgebase, we designed a standard reporting template of individual microbiome data for direct comparison to GutFeelingKB. This type of report can be useful to any scientist, clinician, or patient and can enhance comparison of results from different studies.

## Materials and methods

### Metagenomic sampling and participant statistics

#### Healthy cohort selection and nutritional information

Participants for this study were recruited from the George Washington University (GW) Foggy Bottom campus area through the use of flyers and emails to GW affiliated organizations (selection criterions included in S1 Table). Study participants provided samples and anthropomorphic measurements (included in S1 Table) were collected from healthy people at GW according to a George Washington Institutional Review Board (IRB#011605) approved protocol. At the baseline visit, participants received extensive instructions on how to record their dietary intake (including type, brand, and portion size of every food and beverage consumed on each day throughout the study period) and the time of consumption for each item. Participants then recorded their dietary intake using a seven-day food journal throughout the length of the study. Each participant provided three samples. The food journal was collected at the submission of the final sample, after which the reported 7-day dietary intakes for each subject were entered into the Nutrition Data System for Research (NDSR) [26]. NDSR produces a tabular daily nutrient profile for each day of dietary intake for each individual, which was then added as metadata to the abundance matrices (supplementary table S2 Table). All participants self-reported as ‘healthy’ (participant does not have an obvious or self-declared disease state) at the start of the study and remained healthy throughout.

#### Sampling and sequencing

Fecal samples were collected from healthy volunteers using sterile commode containers at the Milken Institute School of Public Health at the George Washington University (GWSPH). Immediately following collection in ethanol, the fecal samples were stored in a -20° Celsius freezer for a period of up to two weeks, after which, aliquots were placed in longer term storage at -80° Celsius ultra-freezer. Samples were subsequently transported to the sequencing center on dry ice. DNA was extracted using the MoBio PowerFecal DNA Isolation kit25. Double-stranded DNA (dsDNA) concentration and quality was assessed using NanoDrop and the Qubit dsDNA Broad Range (BR) DNA Assay Kit26, respectively. DNA was diluted for library preparation using the Illumina Nextera XT Library Prep Kit, and 1 ng from each sample was fragmented and amplified using Illumina Nextera XT Index Kit primers. Amplified DNA was then cleaned using Agencourt AMPure XP beads, resuspended in buffer, and tested again for concentration, quality, and fragment size distribution on a Bioanalyzer using the Agilent High Sensitivity DNA Kit. DNA libraries were brought to the same nM concentration, pooled, and denatured with 0.2 N NaOH prior to loading on an Illumina MiSeq Reagent Kit v3 and sequencing on the Illumina MiSeq platform. Sequence data FASTQ files were uploaded to BaseSpace (https://basespace.illumina.com/home/index) for sharing and further analysis.

#### Sequence quality assurance

All sequence data were uploaded to the GW High-performance Integrated Virtual Environment (HIVE) [27,28]. Upon initial upload into the system, HIVE automatically conducts a series of quality assurance (QA) computations for each sequence read file and generates figures to display the results. S1 Fig is a compilation of the quality assurance computations done on one read file.

Upon completion of the initial upload for each read file, the resulting quality assurance figures were inspected to ensure that the read file was of adequate quality and did not have any unusual characteristics (such as low-quality score or disproportionate distribution of nucleotides). Reads that had an average Phred quality score of 20 or less were discarded. The nucleotide base distribution was also examined to ensure that no read files had an unusual distribution of bases or a positional quality score below the threshold of 20. S2 Fig is an aggregate of the computations across all samples.

#### Healthy cohort from Human Microbiome Project

In addition to the data generated from sequencing described above, additional data were downloaded and analyzed from the Human Microbiome Project (HMP) [29]. HMP sequence data and metadata are available through NCBI SRA and dbGaP. Fifty fecal metagenomic samples, randomly chosen from HMP Phase I (supplementary table S1 Table) to match approximately the number of samples collected in our study were selected. The samples generated by the HMP project dataset subjects were screened based on stringent criteria listed in their publication and the individuals who passed the screening were considered “healthy” subjects [11].

#### GW and HMP combined data

Sequence and metadata from this study are publicly available through GutFeelingKB (https://hive.biochemistry.gwu.edu/gfkb), and also available from two NCBI-SRA BioProjects (Healthy Human Gut Metagenomics (PRJNA428202), and Effects of non-nutritive sweeteners on the composition of the human gut microbiome (PRJNA487305). For PRJNA487305, only the samples donated prior to intake of non-nutritive sweeteners were used in this study. HMP data were downloaded from NIH Human Microbiome Project (HMP) Roadmap Project (PRJNA43021).

A total of 48 samples from 16 individuals were sequenced in the GW cohort. Each sample resulted in two pair-end read files (for details see S3 Table). Sequence data from these 48 samples along with 50 samples from HMP passed sequence quality checks and were used to develop the baseline microbiota profile. For GW samples 55.55% (± 13.46%) while for HMP 48.29% (± 18.54%) of the reads could not be mapped to any known sequence. There was no need for any computational filtering of human DNA as the MoBio PowerFecal DNA Isolation kit25 was used for GW samples, biochemically removing any host DNA. For the HMP data, all human DNA had been computationally removed before the samples were deposited in dbGaP [11]. Sample and participant information can be seen in Table 1.

**Table 1:** Human Microbiome Project (HMP) and GW participant statistics.

Feature	White	Other	Asian	Black	Male	Female
HMP samples	39	2	7	2	30	20
GW samples	24	0	6	18	21	27

#### Filtered-nt

The Filtered-nt (v5.0) was created from the NCBI-nt file downloaded on May 21st, 2017. A detailed README.md and the code used can be found at https://github.com/GW-HIVE/HIVE-lab/tree/master/Filtered_nt. Both the NCBI-nt (ftp://ftp.ncbi.nlm.nih.gov/blast/db/FASTA) and NCBI taxonomy files (ftp://ftp.ncbi.nlm.nih.gov/pub/taxonomy) were downloaded using the wget command.

Using a curated blacklist file of taxonomy IDs, Filtered-nt was generated based on terms that are contained in the lineage of each taxonomy entry. Taxonomy nodes with terms such as ‘unclassified’, ‘unidentified’, ‘uncultured’, ‘unspecified’, ‘unknown’, ‘vector’, ‘environmental sample’, ‘artificial sequence’, ‘other sequence’ were blacklisted. Child nodes are also automatically removed. The filtered taxonomy list was then used to filter the NCBI-nt sequence file. Filtered-nt and the blacklisted taxonomy IDs along with node names are available for download at https://hive.biochemistry.gwu.edu/filterednt.

### Metagenomic analysis pipeline

The innovative metagenomic analysis pipeline developed includes three software tools and one sequence database (Filtered-nt), organized in a fashion to produce a workflow that ensures an efficient and comprehensive analysis of a large sequence space. The tools are CensuScope [30], HIVE-Hexagon [31], and IDBA-UD [32]. All software tools are integrated in the HIVE platform [27,28] and allow end-to-end analysis of metagenomic sequences.

#### Healthy Human gut microbiome list (GutFeelingKB)

CensuScope [30] is a taxonomic profiling software that randomly extracts a user-defined number of reads and maps them to any size sequence database using BLAST [33]. CensuScope is rapid, accurate, and is not hindered by the size of the reference sequence database. With the non-redundant sequence database’s almost constant exponential increase, CensuScope offers a scalable approach for estimating taxonomic composition of a microbial population. A list of organisms, taxonomy identifiers, and BLAST alignments are provided as the output by CensuScope. A manual evaluation of the CensuScope results for each of the identified organisms was performed to verify that the “hit” represented an authentic match. “Manual evaluation” included the following criteria:

1. Inspection of the match count. The number of matched alignments over the entire computation (over all iterations) had to be > = five out of total 12,500 alignment threshold set by CensuScope. Five was chosen so that there were enough individual alignments to appraise the authenticity of the matches.
2. Confirmation of a justifiable taxonomy assignment. Hits to sequences that lacked a clear taxonomic lineage were excluded and marked for removal from Filtered-nt.
3. Completeness of sequence in GutFeelingKB. Partial sequences, single proteins, or unassembled contiguous sequences were mapped to complete genomes to be included in GutFeelingKB. This is the only way to keep partial sequences from skewing organism abundance results.
4. Organism verification. In order to have confidence in the results, it was necessary to independently verify the biological accuracy of each “hit”. Metadata about the organism was reviewed to verify appropriateness of its presence in the human gut.

Any reference sequence and organism that satisfied these criteria was added to the GutFeelingKB. To extend the usability of this list, available online databases and reference text was used to annotate the organisms [22,34–36]. The NCBI accession numbers from the true positive CensuScope hitlist results were used to obtain the NCBI accession, the RefSeq accession, the NCBI taxonomy ID, the organism name (Scientific Name), the taxonomy id, and the genome assembly IDs. Using the taxonomy ID, the lineage and taxonomic name from the NCBI taxonomy database was retrieved.

Genome to proteome mapping was guided by Representative Proteome Groups (RPGs), a dataset that clusters similar proteomes (https://proteininformationresource.org/rps/). The RPG clusters are calculated based on co-membership in UniRef50 clusters [34] (supplementary tables S4 and S8 Tables). Using the taxonomy ID and the RPG, the corresponding proteome in https://www.uniprot.org/proteomes was identified. From the proteome entry, verification of the Genome Assembly ID match between UniProt, RPG, and NCBI was performed.

In most instances the proteome entry contained some descriptive text about the organism taken from a publication, as well as citations. Such information was added as organism annotation. Additional fields (Resistance to Antibiotic, Susceptibility to Antibiotic, Physical Characteristics) were populated from other sources [36]. Finally, all of the associated DOI and PMIDs for the metadata were added to the final column. It is important to note that many bacteria are closely related and hence have large homologous regions. This can lead to species level misidentification. Although the concept of pan-genome or pan-proteome for closely related bacteria is well accepted [35], it is important to avoid such misidentification for known pathogens. To avoid such false positives of well-known pathogens (S5 Table), they are included only if their abundance is 1% or higher and their alignments have been manually evaluated.

#### Bacterial abundance profile

**Fig 1:**
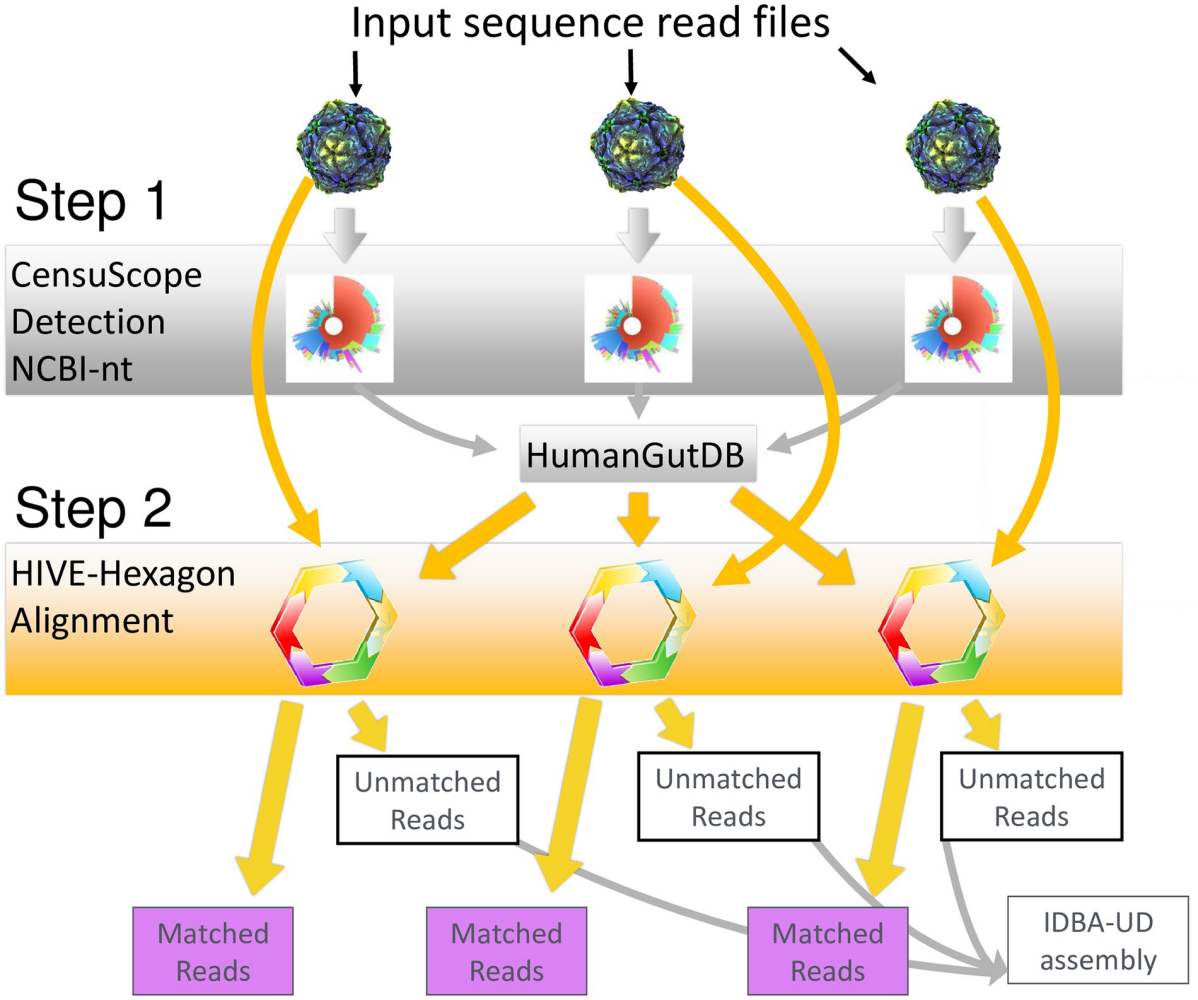
Metagenomic analysis pipeline for 3 samples. Step 1: CensuScope is run for each read file against Filtered-nt. Each of the aligned organism approved by manually check is added to the GutFeelingKB and it is versioned. Step 2: For the final analysis the raw read files are mapped against GutFeelingKB organism sequences using HIVE-hexagon. Outputs are tabulated as relative abundance percentages. Unaligned reads from each sample were assembled using IDBA-UD. Contigs that were over 10,000 nucleotides long had their headers modified to include the following: sample ID, numbered according to length (long to short), and additional metadata data about the participant. These contigs are available as a download at (https://hive.biochemistry.gwu.edu/gfkb).

Fig 1 provides a schematic representation of the workflow. The first step uses CensuScope (a subsampling BLAST algorithm) to identify organisms that are present in the sample. To generate a rapid and accurate taxonomic profile, 2,500 reads are used in each iteration [30] (up to five iterations). This step allows identification of organisms present in a sample. These organisms are added into GutFeelingKB if it is not already present. Next, HIVE-hexagon, a highly specific and sensitive short-read aligner [37], is used to map all of the reads in each sample to GutFeelingKB (created through the use of CensuScope) to obtain the final abundance profiles. It is important to note HIVE-hexagon best match parameter was used. This parameter allows reads to be mapped to the reference (in the case of best matches to more than one reference) which has the greatest number of matches.

#### Metagenomic dark matter

The unaligned reads of each sample were assembled using IDBA-UD [32] and considered as metagenomic dark matter. Only the assembled contiguous sequences (contigs) longer than 10,000 nucleotides were investigated in this experiment. Such a large length threshold was used to ensure that the metagenomics dark matter contigs were truly of biological origin. The gut microbiome of a sample can be represented as the sum of known organisms and organisms represented by the metagenomic dark matter sequences. More specifically, the contigs that were over 10,000 nucleotides in length were tagged with the sample ID and numbered, and metadata data about the participant was added to the header.

These contigs are available as a download at (https://hive.biochemistry.gwu.edu/gfkb) for further analysis and novel primer design.

### Analysis of nutritional metadata and microbial abundance

MaAsLin, an R package that employs a “multivariate statistical framework that finds associations between clinical metadata and microbial community abundance or function” [38] was used to find correlations between bacterial abundance and diet. Intra-host variability was analyzed evaluating the standard deviation of multiple measurements for every patient averaged over all patients. Inter-host variability was computed as a standard deviation of the means of per-host abundance values. To estimate the degree of stability of measurements for bacterial populations in patient samples intra-host vs inter-host variability ratio was computed.

Nutrition to organism abundance correlation was also computed by using a Cosine Similarity Coefficient. The matrix of bacterial strain abundances was variance scaled and zero centered to create comparable distributions of equal variability. Categorical data (such as gender) were turned into numerical values. More specifically, in order to define correlation metrics between features and bacterial composition for the set of individuals, we used Cosine Similarity Coefficient as defined in Formula 1. Cosine Similarity Coefficient of correlation between bacteria (j) and feature (k) is computed as the sum product of j^th^ Bacteria (Bj) abundance for patient i and k^th^ Feature (Fk) of patient i.

$${Correlation}_{j,k}=\sum_{i=1}^{N}B_{i,j}\times F_{i,k}$$

A Cosine Similarity of around 1 indicates a strong correlation, -1 indicates a strong anti-correlation, 0 is no correlation with 0.7 being considered the marginal threshold for evidence of some degree of correlation [39,40].

## Results and discussions

### Filtered NCBI-nt (Filtered-nt)

NCBI nucleotide sequence collection (NCBI-nt) is the most comprehensive collection of DNA sequences [22], but many sequences present in NCBI-nt do not provide enough relevant information or correct metadata (e.g. sequences with taxonomy placement such as environmental, unclassified, synthetic sequences, unidentified sequences etc.). A great number of the sequences available in NCBI-nt are also artificial. Reads mapped to such sequences do not provide any valuable biological information in a clinical setting and hence are not useful in understanding the microbial composition of a sample. The version of NCBI-nt used to create our Filtered-nt (v5.0) initially contained 42,439,338 sequences and the taxonomy file contained 1,601,859 scientific names. After removal of 250,610 blacklisted taxonomy IDs (supplementary table S6 Table) pertaining to 7,499,592 sequences the Filtered-nt contained 34,939,806 sequences. The Filtered-nt is ideal for comprehensive metagenomic analysis that relies on a best sequence hit.

Most studies use genomes from known gut bacteria as a truncated reference database [18,30,41,42] and hence would not be able to detect organisms that are not present in their reference database. The use of our Filtered-nt offers surety that the entirety of the known sequence space is covered while excluding the *in-silico* sequence space and the uncultured/unclassified sequence space.

### Healthy fecal microbiome

#### GutFeelingKB—A reference list for healthy human gut organisms

GutFeelingKB is a compilation of highly curated data and metadata associated with organisms identified as present in the samples we analyzed. GutFeelingKB consists of 157 organisms which fall into sixty distinct genera, as seen in S2 Table which is arranged by species. The full table can be downloaded at https://hive.biochemistry.gwu.edu/gfkb. Members of the Firmicutes and Bacteroidetes phyla make up a majority of the bacterial species that were present in the human intestinal microbiota. A total of 155 bacterial and 2 archaeal organisms were identified in healthy samples. In summary, the healthy human gut microbiome consists of 8 phyla, 18 families, 23 classes, 38 orders, 59 genera and 109 species. 63 (40%), 32 (20%) and 31 (19.7%) members belongs to Firmicutes, Actinobacteria and Bacteroidetes, respectively which make up a majority of the bacterial species. More than half of Firmicutes are members of the Clostridia (20.3%) class, which is the most abundant class, followed by Bacteroidia (18.5%), Bifidobacteriales (16.6%), Enterobacterales (14%) and Lactobacillales (14%). All of members of Clostridia in the samples are members of Clostridiales order and all of Bacteroidia belongs to Bacteroidales, these two are the most abundant orders. There are 27 organisms which are members of Bifidobacteriaceae family, and 26 of them belongs to *Bifidobacterium longum*, which is the most abundant species.

With respect to core species concept, 84 out of 109 organisms are present in all of the samples (Table 2). These 84 could feasibly be a core organisms list for the human gut, but for this paper the focus is on creating a comprehensive list of organisms found in healthy individuals. Supplementary file S3 Fig shows the exponential decrease of new organisms identified in each additional sample. Supplementary file S7 Table provides a list of 129 organism clusters that are similar to the organisms (similarity based on computational clustering of proteomes at 75% co-membership threshold [34]) in GutFeelingKB. This could serve as a supplement the GutFeelingKB to avoid the misidentification of highly-similar organisms. All these 863 organisms comprise an expanded set of microbes that can be present in a healthy human gut.

**Table 2:** List of 109 baseline species and their GenBank accessions found in healthy human gut.

Organism name	GenBankAC	Organism name	GenBankAC	Organism name	GenBankAC
Acidaminococcus fermentans (Bac/Firmicute) (100^1^;0.04^2^)	CP001859	Clostridium saccharolyticum (Bac/Firmicute) (100;0.24)	CP002109, FP929037	Odoribacter splanchnicus (Bac/CFB_bac) (100;1.12)	CP002544
Acidaminococcus intestine (Bac/Firmicute) (100;0.09)	CP003058	Coprococcus catus (Bac/Firmicute) (100;0.37)	FP929038	Ornithobacterium rhinotracheale (Bac/CFB_bac) (100;0.11)	CP006828
Acidovorax sp KKS102 (Bac/Beta-proteo) (100;0.01)	CP003872	Coprococcus sp ART55/1 (Bac/Firmicute) (100;0.68)	FP929039	Oscillibacter valericigenes (Bac/Firmicute) (100;0.05)	AP012044
Adlercreutzia equolifaciens (Bac/ActnBac) (100;0.07)	AP013105	Cutibacterium acnes (Bac/ActnBac) (100;0.004)	CP003084	Paenibacillus sabinae (Bac/Firmicute) (100;0.01)	CP004078
Akkermansia muciniphila (Other Bacteria) (91.84;0.70)	CP001071	Eggerthella lenta (Bac/ActnBac) (100;0.04)	CP001726	Paeniclostridium sordellii (Bac/Firmicute) (100;0.02)	LN679998, LN681234
Alistipes finegoldii (Alistipes finegoldii) (100;1.27)	CP003274	Eggerthella sp. YY7918 (Bac/ActnBac) (100;0.01)	AP012211	Parabacteroides distasonis (Bac/CFB_bac) (100;2.30)	CP000140
Alistipes shahii (Bac/CFB_bac) (100;1.75)	FP929032	Enterococcus faecium (Bac/Firmicute) (100;0.04)	CP003351, CP006620, CP006030	Parvimonas micra (Bac/Firmicute) (100;0.01)	CP009761
Anaerococcus prevotii (Bac/Firmicute) (100; 0.003)	CP001708	Enterococcus hirae (Bac/Firmicute) (96.94;0.004)	CP003504	Porphyromonas asaccharolytica (Bac/CFB_bac) (98.98;0.01)	CP002689
Anaerostipes hadrus (Bac/Firmicute) (100;0.55)	FP929061	Escherichia coli (Bac/Gamma-proteo) (100;1.87)	CP009859, CP010816, CP000948, CP001637, CP000970, CP000243, CP009166, CP002291, CP003297, CP007394, AP009378, AE014075, CP010371, CP002729, CP007799, CP001396, CP009789, CP004009, CP007390, FN649414, CP009167, HG941718	Porphyromonas gingivalis (Bac/CFB_bac) (100;0.01)	AP009380
Bacillus methanolicus (Bac/Firmicute) (100;0.01)	CP007739	Escherichia coli O104:H4 (Bac/Gamma-proteo) (96.94;0.04)	CP004009	Prevotella dentalis (Bac/CFB_bac) (100;0.08)	CP003368, CP003369
Bacteroides cellulosilyticus (Bac/CFB_bac) (100;3.38)	CP012801	Escherichia coli O83:H1 (Bac/Gamma-proteo) (95.92;0.06)	CU651637	Prevotella denticola (Bac/CFB_bac) (98.98;0.04)	CP002589
Bacteroides dorei (Bac/CFB_bac) (100;17.44)	CP007619, CP009057	Ethanoligenens harbinense (Bac/Firmicute) (100;0.01)	CP002400	Prevotella intermedia (Bac/CFB_bac) (100;0.07)	AP014597, CP003502, CP003503, AP014598
Bacteroides fragilis (Bac/CFB_bac) (100;3.47)	FQ312004, CR626927, AP006841, AP006842, CR626928	Eubacterium eligens (Bac/Firmicute) (100;0.65)	CP001104, CP001105, CP001106	Prevotella melaninogenica (Bac/CFB_bac) (100;0.24)	CP002122, CP002123
Bacteroides helcogenes (Bac/CFB_bac) (100;0.50)	CP002352	Eubacterium limosum (Bac/Firmicute) (100;0.03)	CP002273	Prevotella ruminicola (Bac/CFB_bac) (100;0.06)	CP002006
Bacteroides ovatus (Bac/CFB_bac) (100;7.72)	CP012938	[Eubacterium] rectale (Bac/Firmicute) (100;6.21)	FP929042, FP929043, CP001107	Prevotella sp oral taxon 299 (Bac/CFB_bac) (100;0.06)	CP003666
Bacteroides salanitronis (Bac/CFB_bac) (100;0.48)	CP002530	[Eubacterium] siraeum (Bac/Firmicute) (100;0.75)	FP929044, FP929059,	Raoultella ornithinolytica (Bac/Gamma-proteo) (100;0.01)	CP004142
Bacteroides sp. CAG:98 (Bac/CFB_bac) (100;8.89)	CP008741	Faecalibacterium prausnitzii (Bac/Firmicute) (100;3.52)	FP929045, FP929046	Roseburia hominis (Bac/Firmicute) (100;0.69)	CP003040
Bacteroides thetaiotaomicron (Bac/CFB_bac) (100;3.78)	AE015928, AY171301	Faecalitalea cylindroides (Bac/Firmicute) (100;0.15)	FP929041	Roseburia intestinalis (Bac/Firmicute) (100;1.15)	FP929049, FP929050
Bacteroides vulgatus (Bac/CFB_bac (100;14.99)	CP000139	Fermentimonas caenicola (Bac/CFB_bac) (100;0.01)	LN515532	Rubinisphaera brasiliensis (Bac/Plnctmy) (70.41;0.0002)	CP002546
Bacteroides xylanisolvens (Bac/CFB_bac) (100;4.92)	FP929033	Gardnerella vaginalis (Bac/ActnBac) (91.84;0.002)	CP001849	Ruminococcus bicirculans (Bac/Firmicute) (100;2.54)	HF545616, HF545617
Barnesiella viscericola (Bac/CFB_bac) (100;0.33)	CP007034	Gordonibacter pamelaeae (Bac/ActnBac) (100;0.03)	FP929047	Ruminococcus bromii (Bac/Firmicute) (100;0.83)	FP929051
Bifidobacterium adolescentis (Bac/ActnBac) (97.96;0.46)	CP007443, CP010437, AP009256	Haemophilus parainfluenzae (Bac/Gamma-proteo) (100;0.10)	FQ312002	Ruminococcus champanellensis (Bac/Firmicute) (100;0.04)	FP929052
Bifidobacterium animalis (Bac/ActnBac) (100;0.03)	CP009045	Intestinimonas butyriciproducens (Bac/Firmicute) (100;0.24)	CP011307	Ruminococcus sp SR1/5 (Bac/Firmicute) (100;0.68)	FP929053
Bifidobacterium bifidum (Bac/ActnBac) (100;0.31)	CP010412, CP001840, CP002220, CP001361	Klebsiella aerogenes (Bac/Gamma-proteo) (91.84;0.01)	FO203355, CP002824	Ruminococcus torques (Bac/Firmicute) (100;0.97)	FP929055
Bifidobacterium breve (Bac/ActnBac) (97.96;0.01)	CP006715, CP006713	Klebsiella michiganensis (Bac/Gamma-proteo) (93.88;0.002)	CP004887	Sphingobacterium faecium (Bac/CFB_bac) (95.92;0.04)	LK931720
Bifidobacterium dentium (Bac/ActnBac) (85.71;0.01)	AP012326	Klebsiella pneumoniae (Bac/Gamma-proteo) (88.78;0.01)	CP009208	Streptococcus mitis (Bac/Firmicute) (100;0.02)	FN568063
Bifidobacterium kashiwanohense (Bac/ActnBac) (100;0.13)	AP012327, CP007456	Klebsiella variicola (Bac/Gamma-proteo) (90.82;0.01)	CP001891	Streptococcus parasanguinis (Bac/Firmicute) (100;0.04)	CP002843, CP003122
Bifidobacterium longum (Bac/ActnBac) (100; 0.74)	AP014658, CP002286, CP011964, CP000605, LN824140, AP010890, AP010889, AP010888, CP002010, FP929034, CP006741, CP002794, CP009072	Lachnoclostridium phytofermentans (Bac/Firmicute) (100;0.09)	CP000885	Streptococcus pasteurianus (Bac/Firmicute) (100;0.02)	AP012054
Bifidobacterium thermophilum (Bac/ActnBac) (100;0.005)	CP004346	Lactobacillus acidophilus (Bac/Firmicute) (93.88;0.003)	CP005926	Streptococcus salivarius (Bac/Firmicute) (100; 0.09)	CP009913, FR873482, CP002888, FR873481
Blautia obeum (Bac/Firmicute) (100;0.51)	FP929054	Lactobacillus paracasei (Bac/Firmicute) (100;0.01)	AP012541	Streptococcus sp I-P16 (Bac/Firmicute) (100;0.01)	CP006776
butyrate-producing bacterium SM4/1 (Bac/Firmicute) (100;0.13)	FP929060	Lactobacillus rhamnosus (Bac/Firmicute) (92.86;0.01)	CP003094	Streptococcus suis (Bac/Firmicute) (100;0.02)	CP000837
butyrate-producing bacterium SS3/4 (Bac/Firmicute) (100;0.36)	FP929062	Lactobacillus ruminis (Bac/Firmicute) (100;0.16)	CP003032	Streptococcus thermophilus (Bac/Firmicute) (100;0.05)	CP000024, CP000419, CP006819
Campylobacter coli (Bac/Delta-Epsilon-proteo) (100;0.01)	CP007180	Lactococcus lactis (Bac/Firmicute) (96.94;0.01)	CP006766	Tannerella forsythia (Bac/CFB_bac) (100;0.06)	CP003191
Campylobacter hominis (Bac/Delta-Epsilon-proteo) (97.96;0.003)	CP000776	Leuconostoc citreum (Bac/Firmicute) (93.88;0.003)	DQ489736	Treponema succinifaciens (Other Bacteria) (100;0.03)	CP002631
Candidatus Methanomassiliicoccus intestinalis (Arch/Euryar) (34.69;0.01)	CP005934	Mageeibacillus indolicus (Bac/Firmicute) (100;0.01)	CP001850	Veillonella parvula (Bac/Firmicute) (100;0.05)	CP001820
Citrobacter freundii (Bac/Gamma-proteo) (84.69;0.02)	CP007557	Megamonas sp Calf98-2 (Bac/Firmicute) (100;0.02)	FP929048		
Clostridioides difficile (Bac/Firmicute) (1.02;2.10)	CP003939, CP010905	Methanobrevibacter smithii (Arch/Euryar) (39.80;0.07)	CP000678		

^1^ Percentage of samples this organism is present in.

^2^ Average percent relative abundance of this organism.

Several researchers have focused on the reference genes of the gut microbiome rather than organisms [43,44], but organisms have their own clinical significance in treatment. When Yatsunenko et al. analyzed 531 healthy samples from Venezuela, rural Malawi and US metropolitan areas and mapped their reads to 126 microbial species, they found Fusobacteria that were not mapped to our list. On the other hand, Spirochaetes, Planctomycetes identified in this study were not shown in their list [45]. Of the organisms reported in their study, forty genera map to our list at the species level. Unmapped species include organisms such as Actinomyces odontolyticus, Bacteroides capillosus, Bacteroides uniformis. Nishijima et al. identified 26 major genera in healthy Japanese [46]. Twenty of the 26 genera they listed mapped to the list from this study, the unmapped genera belong to existing GutFeelingKB families and are *Dorea*, *Dialister*, *Succinatimonas*, *Butyrivibrio*, *Coriobacteriaceae*, and *Phascolarctobacterium*. Qin et al. grouped 66 clusters representing cognate bacterial species for healthy and liver cirrhosis patients [47], and the lowest taxonomy level of cluster in this study is strain. Thirty-six of these clusters map to GutFeelingKB in the taxonomy levels higher than species and all of them map to existing GutFeelingKB families. These studies of healthy microbiome diversity from around the world suggest there is significant regional heterogeneity in the health gut microbiome at species/strain level, but reasonable consistency at higher taxonomic levels.

In a study conducted to demonstrate the feasibility of accurate detection of clinically relevant prokaryotic targets [20], Almonacid et al. showed that it was practical to identify 28 specific targets (14 species and 14 genera) based on sequencing of the 16S rRNA marker gene, which is an important clinical application when considering the cost of a test. Adapting one of their supplementary files (https://doi.org/10.1371/journal.pone.0176555.s003), we were able to determine that 75 of the organisms listed in GutFeelingKB can be mapped to Almonacid et al.’s clinical targets. The mapping was done by identifying UniProt Proteome IDs [35] and the RPG [34] cluster that best matched the organism described. If the organism was not present in GutFeelingKB, then we reference our supplementary file S7 Table to see if the organism was present in one of the RPG clusters that are represented by the organisms in GutFeelingKB. The results of our mapping against this study is included as a supplementary table (S11 Table).

Twelve of the 28 clinical targets were unable to be resolved with GutFeelingKB. Only two of these were genera, with the rest being a species level classification. Both of the genera are positively associated with abnormal GI states (*Salmonella* with Diarrhea [48] and *Fusobacterium* with Irritable Bowel Syndrome [49]. Of the ten species, five were positively associated with diarrhea or IBD (*Vibrio cholerae*, *Salmonella enterica*, *Streptococcus sanguinis*, *Desulfovibrio piger*, *and Anaerotruncus colihominis*). Only one of the species listed, *Collinsella aerofaciens*, did not have a reference proteome (https://www.uniprot.org/help/reference_proteome).

It is expected that while other studies will find additional organisms, GutFeelingKB can provide a reference list and abundance information that can provide a starting point for comparative analysis of samples from healthy individuals from around the world and can also help better understand observed differences due to disease, therapy, and diet.

#### Organism abundance in individual samples

Data interoperability is a perennial challenge in bioinformatics [50]. This problem is further magnified when considerations are made for data from samples collected in distant locations at different times. In the case of HMP, sampling was done in Houston, TX and St. Louis, MO during 2008–2012. All GW samples were collected from the DC Metro Area in 2016. One way to test the compatibility of these data sets was to run a Between-Class Analysis (BCA) on all samples from each of the projects. Data from our three, separate projects fell into the expected three classic enterotypes [51] instead of clustering by project set (S4 Fig). Had the data clustered by project, sampling location, or year, they may not have been compatible for inclusion in the same database. However, we believe that these data do not show a sampling bias and can be leveraged for joint analysis. The sample and participant information are presented in Table 1.

**Fig 2:**
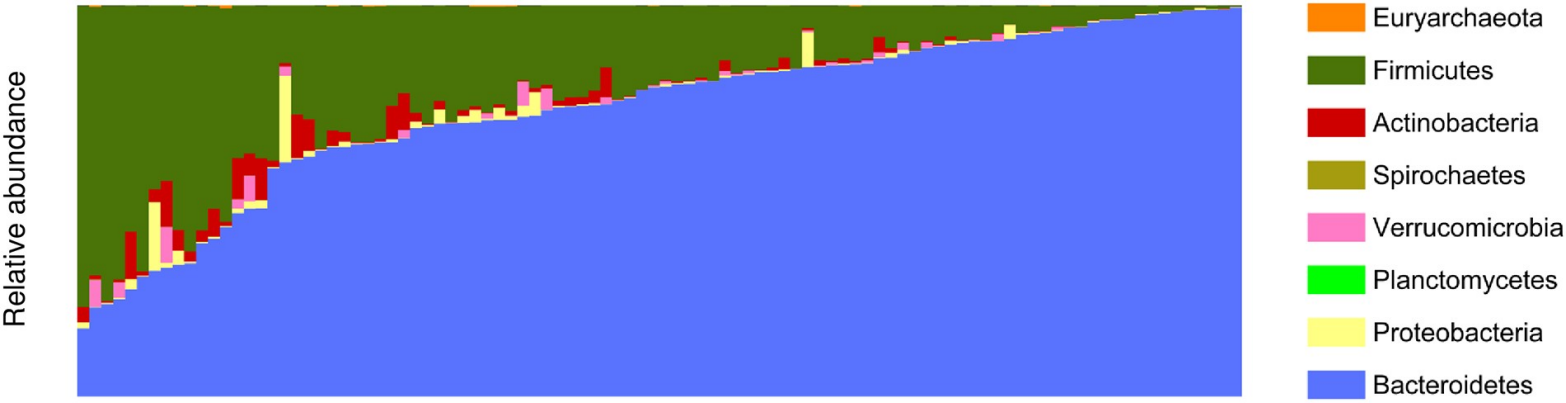
Stacked bar plot of phylogenetic composition of all microbiome taxa in this study collapsed at the phyla level in fecal samples. Green bars represent *Firmicutes* and the blue represent *Bacteroidetes*, the two most abundant bacterial families. For aesthetic purposes the samples (n = 98, bottom) were sorted according to their composition of *Bacteroidetes* and *Firmicutes* to demonstrate how the baseline gut microbiome results from this study could be used in conjunction with results from past studies.

Many studies have focused on higher taxonomy nodes, providing little abundance information about specific species or strains. Fig 2 shows the abundance of phyla to highlight how baseline gut microbiome results from this study can be used to compare results from past studies. Abundance sheet with the lowest taxonomy node broken down to the strain level, where applicable, is provided so that other scientists can use the results for comparison purposes. Average abundance, standard deviation, maximal and minimal abundance excluding the organisms with the 0% abundance (S9 Table) provides additional metrics. In terms of average abundance of organisms, 4 phyla have abundance above 1%, these are Actinobacteria (1.82± 3%), Bacteroidetes (73.13 ± 22.16%), Firmicutes (22.2 ± 18.66%) and Proteobacteria (2.15 ± 10.39%). Bacteroidia (72.97 ± 22.14%) under Bacteroidetes, Actinobacteria (1.67 ± 2.94%) under Actinobacteria, Gammaproteobacteria (2.12 ± 10.38%) under Proteobacteria, Clostridia (21.35 ± 17.87%) under Firmicutes are the only four classes that have average abundance larger than 1%. Bacteroidaceae (65.58 ± 21.84%) is the most abundant family, followed by Lachnospiraceae (11.46 ± 11.06%) and Ruminococcaceae (8.38 ± 10.48%). Odoribacteraceae, Rikenellaceae, Bifidobacteriaceae, Enterobacteriaceae and Tannerellaceae are the five other families with abundance above 1%. *Bacteroides* is the most abundant genus in human gut microbiome (65.58 ± 21.84%) with sample SRS016585 having the smallest abundance (0.37%) while SRS013215 has the largest abundance (98.82%). *Bacteroides* includes 9 species and 7 of them have abundance greater than 1%. *Bacteroides dorei* is the most dominant species with a 17.44 ± 8.74% abundance.

Out of 98 samples analyzed, only 53 samples had archaea. Bacteroidetes, Proteobacteria, Spirochaetes, Actinobacteria, Firmicutes phylum are present in all samples. The abundance of Bacteroidetes is greater than 10% in 97 of 98 samples. *Bacteroides* is present in all the samples with an abundance ranging from 0.37% to 98.82%. Within *Bacteroides*, *Bacteroides fragilis* is present in all the samples. The range of *Bifidobacterium* abundance in all the samples ranges from 0.004% to 12.21%, *Bifidobacterium longum* abundance from 0.003% to 10.30% and *Bifidobacterium bifidum BGN4* strain is present in 96 of 98 samples. A total of 84 out of 109 species are present in all of the samples.

It has been shown that *Bacteroides* is the most abundant genus in Spain, China, Sweden, US, Denmark and France from samples collected from healthy individuals [46]. *Bacteroides* maintain a generally beneficial relationship with the host when retained in the gut but can also be opportunistic pathogens. When they escape the gut environment, they can cause significant pathology, including bacteremia and abscess formation in multiple body sites [52]. *Bacteroides fragilis* protects animals from colitis induced by *Helicobacter hepaticus*, a commensal bacterium with pathogenic potential [53]. A large proportion of the *B*. *fragilis* genome is responsible for carbohydrate metabolism, including the degradation of dietary polysaccharides [54]. *Bifidobacterium* has been reported to be present in almost all healthy human fecal samples. Members of *Bifidobacterium* are among the first microbes to colonize the human gastrointestinal tract and are believed to exert positive health benefits on their host [55]. Many species of *Bifidobacterium* are commonly used as probiotics due to their health promoting properties [56]. Certain *Bifidobacterium longum* strains have been used as probiotics against enterohemorrhagic *Escherichia coli* infection due to the production of acetate, a short chain fatty acid, which upregulates a barrier function of the host gut epithelium [57]. In general, they are able to survive in particular ecological niches due to competitive adaptations and metabolic abilities through colonization of specific appendages. There are 12 strains under *Bifidobacterium longum* species. One strain, BBMN68 has been isolated from the feces of a healthy centenarian living in an area of BaMa, Guangxi, China, known for longevity [58]. Another strain of *Bifidobacterium*, BGN4, was shown to prevent CD4(+) CD45RB (high) T-cell mediated inflammatory bowel disease by inhibition of disordered T cell activation in BGN4-fed mice [59]. Despite the well-established health benefits, the molecular mechanisms responsible for these traits remain to be elucidated.

Some potential pathogenic species appear in healthy samples in this study and the samples collected by Yatsunenko et al. [45]. *Streptococcus mitis*, a strain that can cause severe clinical symptoms in cancer patients [60] was also identified. It is likely that organisms such as *S*. *mitis* are opportunistic pathogens. There are several strains of *Escherichia coli*, for which the majority of strains are generally considered a harmless intestinal inhabitant. *E*. *coli* is one of the first bacterium to colonize human infants and is a lifelong colonizer of adults [61], although pathogenic strains of *E*. *coli* have been implicated in the etiology of health problems such as Crohn’s disease and ulcerative colitis [62].

### Dietary data and nutrient correlative analysis

**Fig 3:**
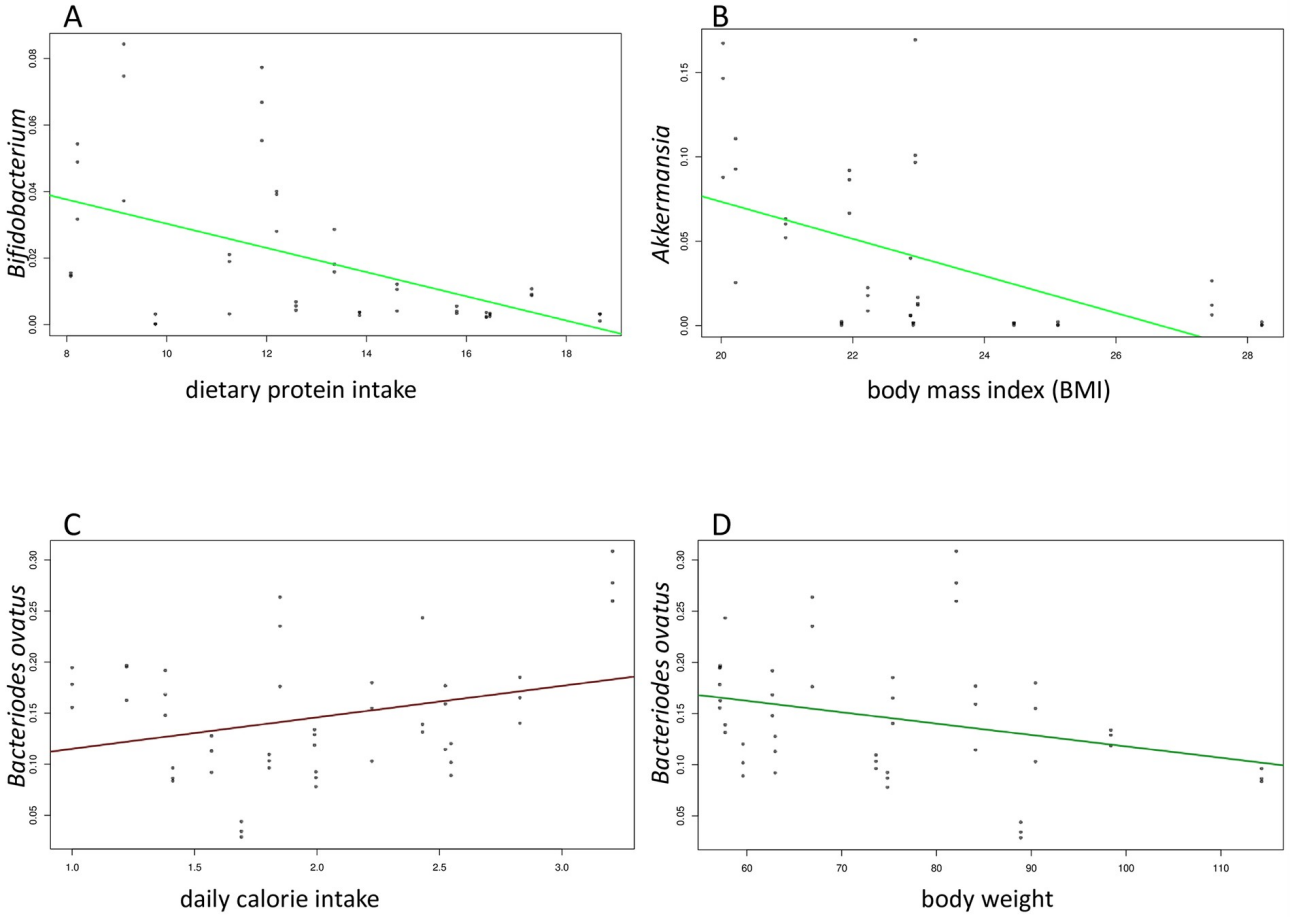
Correlation between bacterial organism and nutrient data. (A) *Bifidobacterium* is positively correlated with dietary protein intake, specifically vegetable protein, present in vegetables such as broccoli, brussel sprouts, beans, peas, asparagus and beans. (B) *Akkermansia* is positively associated with body mass index (BMI). (C) *Bacteriodes ovatus* is positively correlated with daily calorie intake. (D) *Bacteriodes ovatus* is negatively correlated with body weight.

**Fig 4:**
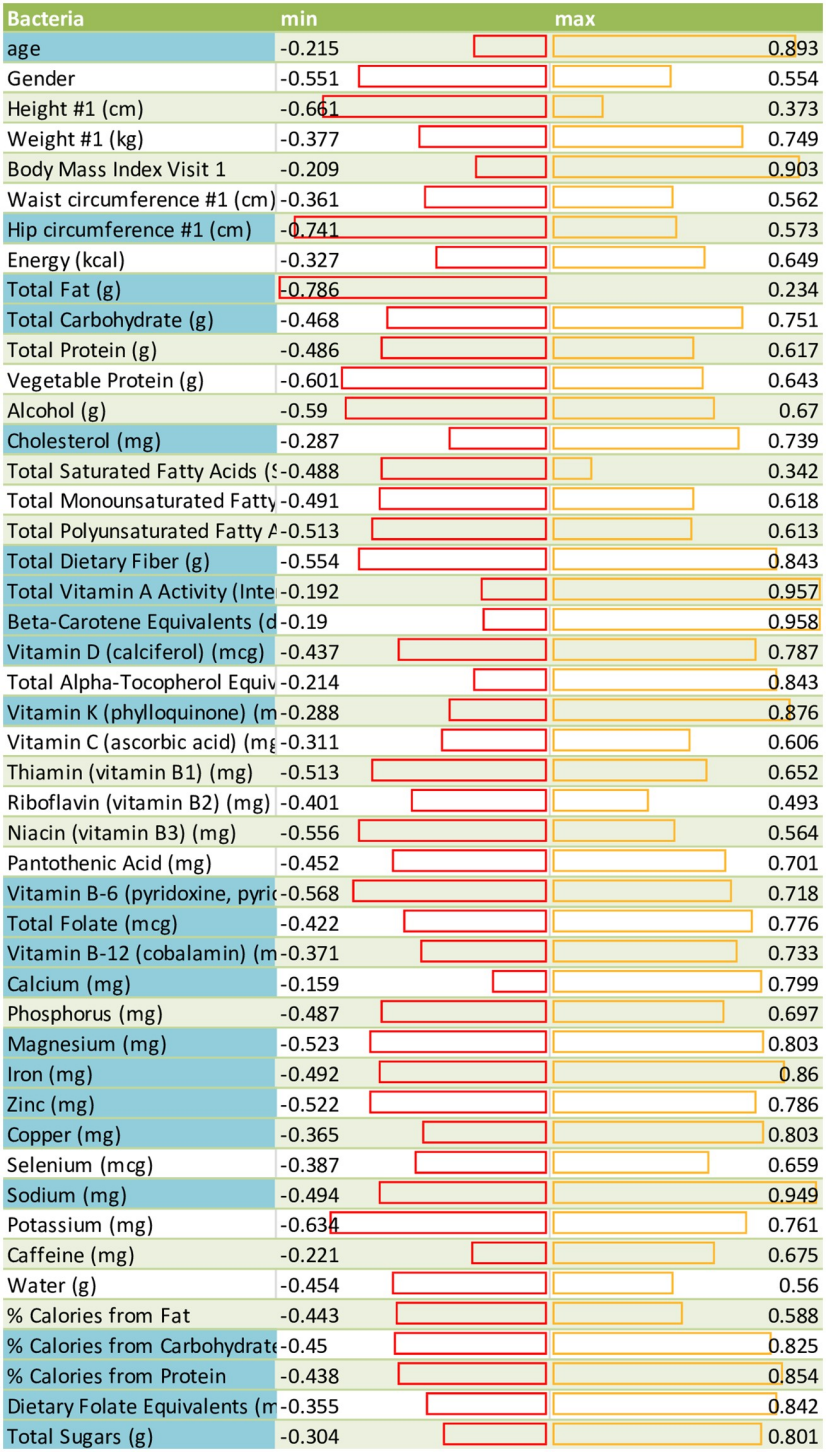
The range of correlation for all features that have been measured for each of the GW samples. Each line is a graph of the min and max values using a Cosine Similarity coefficient correlation. A positive value means strong correlation, and a negative value means strong anticorrelation, whereas zero means absolutely no correlation. Given the size of sample pool of 16, 0.7 is taken as the marginal threshold for evidence of some degree of correlation. Each feature that had a correlation with any organism is highlighted in blue. For example, some characteristics such as fat intake have anticorrelation with members of *Campulobacter jejuni* and Eubacterium family.

In comparing bacterial species to nutrient data using MaAslin, several interesting patterns were observed. *Bifidobacterium* was positively correlated with dietary protein intake (Fig 3a), specifically vegetable protein, as well as dietary fiber, specifically soluble fiber, present in vegetables such as broccoli, brussel sprouts, beans, peas, asparagus and beans, which also contain vegetable protein. *Akkermansia* (Fig 3b) was shown to be positively associated with saturated fat intakes and is negatively correlated with total polyunsaturated fatty acids (PUFA). Not surprisingly, it was also positively correlated with linoleic acid, as this particular omega-6 PUFA is found abundantly in oils (e.g. soybean oil, vegetable oil) used in processed food. *Bacteriodes ovatus* was positively correlated with daily calorie intake (Fig 3c), as well as body weight (Fig 3d), and waist circumference. The table of results (see supplementary file S10 Table) demonstrates the range of correlation for features that have been measured. Cosine Similarity Coefficient analysis (see supplementary file S11 Table) identified correlation for features and organisms with the observations similar to MaAslin. For example, characteristics such as fat intake and BMI correlate with members of *Akkermansia*. Similarly, the impact of Vitamin A or beta carotenes has positive inductive correlation across all the Bifidobacterium (Fig 4).

As microbiome science moves closer to the clinic, it will be imperative both to have tools for analysis and the quick understanding of a microbial population. It is envisioned that such analyses provide the foundation for clinical reporting. While each organism in an entire microbiome sample isn’t immediately actionable, it does allow for both the close tracking of microbial modulation and the better understanding of how the microbiome tracks with health states and therapy. This will be further applicable as evidence-based medicine approaches microbiome science, and microbiome science becomes as important to clinical treatment as genomic medicine. Preliminary microbiome analyses are increasingly yielding interesting results in complex diseases such as cancer. For example, in colorectal cancer patients, carcinoma-enriched bacteria, *B*. *massiliensis*, *B*. *dorei*, *B*. *vulgates*, *Parabacteroides merdae*, *A*. *finegoldii* and *B*. *wadsworthia*, is positively correlated with red meat consumption and negatively correlated with fruit and vegetables consummation [63]. It is expected that as the number and size of these studies increase, the need for baseline human gut microbial profile in healthy people and standard reporting template will become essential.

#### Contigs from unaligned reads (microbial dark matter)

On average, 50% of the reads from an individual sample could not be aligned to any sequence in Filtered-nt. These unaligned reads were assembled into contigs. Previous work has shown that creation of contigs from unaligned short reads can be used to better understand the actual sequence space represented in metagenomics samples [64]. This “microbial dark matter” remains to be elucidated. Using BLAST against NCB-nt sequences did not yield any significant matches. Given that the average protein-coding density of bacterial genomes is 87% with a typical range of 85–90% [65], and the organisms in our reference list range in size from 1.89–6.17 Mb, contigs less than 10Kb were excluded. This value would mean that any single sequence would cover at the very least 0.16% of the organism’s genome, or 0.19% of an organism’s coding region. The goal here was to reduce the number of false positive contigs. Using this approach unaligned reads were assembled into 1,467,129 contigs of which 46,095 have a length greater than 10Kb. After building the contigs, sequences greater than 10,000 nucleotides were saved into the same file, and each header was formatted to indicate the sample number, gender, age, and ethnicity of the source. The file is available for download at https://hive.biochemistry.gwu.edu/prd/gfkb//content/unalignedContigsGFKB-v2.0.fasta. These contigs are ideal for new primer design for detailed analysis of the gut microbiome.

The unaligned reads were used for contig assembly post-alignment to minimize risk of loosing informative contigs to consensus sequences which may map partially to the organisms in GutFeelingKB. This was confirmed in an experiment where all the reads were assembled first, then the contigs were mapped to the genomes from GutFeelingKB. This step resulted in much smaller number of contigs over 10 KB. Most likely some of the dark matter contigs are from bacteriophages. Using the pre-assembly method, one could potentially identify novel bacteriophages and associate the phage with their host organism.

### FecalBiome Reporting Template

**Fig 5:**
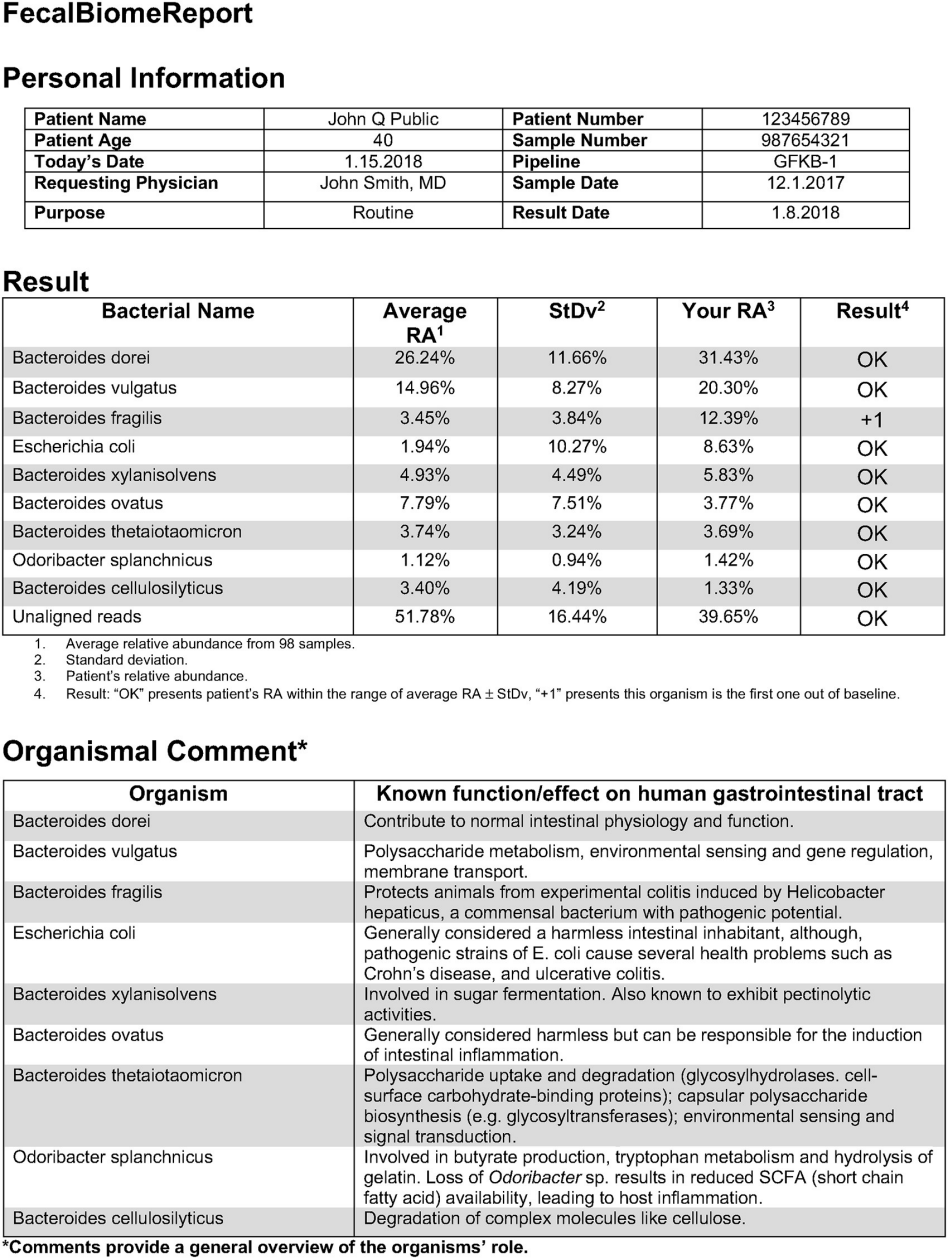
FecalBiome Reporting Template. Personal Information section of the report contains information about the individual who had a sample sequenced, as well as the individual who ordered the sequence. It contains information about the pipeline used for analysis, as well as the sample number for ease of retrieval. Result section contains microbes representing the most abundant organisms which comprise the top 50% of inhabitants. Organismal Comment section includes information from the GutFeelingKB which pertains to the potential function of that organism.

The known effects of the microbiome on health status are growing rapidly and have already spawned FDA approved products at various biotech firms [66]. Some firms have even begun to report microbial composition data to consumers. The formats and parameters for generation of these reports are non-standardized, limiting their research value. It is necessary to standardize the way that the microbiome is discussed in research and, eventually, in the clinic; the earlier this standardization occurs, the more effective it will be as microbiome science becomes a tool for general research and microbiome medicine moves as close to clinic as genomic medicine. Since there is a need for a cycle moving from bench to bedside and back again, there is value in building a clinical-style report on top of a research tool with the ability to easily cross between the two [67]. In that vein, FecalBiome Template is presented (Fig 5)—a general reporting template for microbiome research. It is composed of three domains: Sample, Patient, and Result; these results are drawn from information from a given microbiome sample which is then compared to the contents of the GutFeelingKB. The template was drafted in the spirit of comprehensive metabolic panel (CMP) lab test (https://www.accesalabs.com/downloads/quest-lab-test-sample-report/Comprehensive-Metabolic-Panel-Test-Results.jpg; https://medlineplus.gov/ency/article/003468.htm). This report is also intended to serve as a snapshot of a research project, allowing colleagues and collaborators across labs to share high level information in a rapid manner. It is not uncommon for sample collection, sequencing, and analysis to happen at different locations with different research groups each having a stake in the data produced. In a research setting, the template can serve as a coversheet for shared data, accompanying sequence data to give collaborators a look at their data without having to write scripts for visualizations. This report is designed to be generalizable to any human microbiome. Researchers and clinicians should determine a threshold for the number of organisms reported depending on the circumstances of their investigation. Recommendation from this study is reporting of the organisms that comprise the top 50% (sorted based on abundance) of identified microbes from an individual’s sample. Any threshold of organisms to report in the domains can be set by the user to fit their purposes. Information about sample abundance, average abundances for a microbe, as well as information about those microbes from the literature is included on this report.

As a test case, one sample was taken from the set to determine where it fell relative to the baseline gut microbial population to show the potential clinical application of this technology. The final column in the result table includes information about whether a given population of microbes falls within the range expected based on the sample space included in GutFeelingKB. The report does not include an explanation for what a particular result means, as it is premature to tie a specific microbe to phenotype in cases other than infectious disease, and any result falls to the purview of the requesting physician. With more information on the role of the microbiome and its constituent microbes, it will become more feasible to contrast where a sample from an individual lies within the spectrum of healthy or dysbiotic microbes abundance.

All relative abundances were calculated for the individual datasets before quantifying the relative min, relative max, mean, median, and standard deviation (Fig 3). These statistics were then transformed into one cohesive report that merged the range, mean, median, and standard deviation. The statistics were further collapsed by family to generate an overall report that models a complete metabolic profile. The top most abundant families (Akkermansiaceae, Bacteroidaceae, Enterobacteriaceae, Rikenellaceae, and Ruminoccocaceae) had a relative max of 8.03, 12.13, 10.99, 6.89, and 6.31 percent of relative abundance, respectively. This is not surprising considering the Rikenellaceae family is indicative of good gastrointestinal health [68]. Akkermansiaceae is linked to lower rates of obesity and associated metabolic disorders [69]. Bacteroidaceae and Enterobacteriaceae can be linked to acute infective processes but are otherwise symbionts [70,71], and Ruminococcaceae is known to break down complex carbohydrates especially in people with carb heavy diets [72]. FecalBiome and the underlying GutFeelingKB can have high value to clinicians who hope to assess the gut microbial status of their patients. The goal of the database and report is to connect lab results with outcomes. At present, most known microbiome disease associations are a type of severe dysbiosis caused by a kind of potentially pathogenic bacteria–the canonical infectious pathogens such as *Helicobacter pylori*, *Vibrio cholerae* and others. By determining what species or strain correlate with good or bad outcomes, this type of research could aid clinicians in developing strategies for valuable evidence-based treatments.

## Conclusion

The metagenomic analysis workflow described in this study involves a sub-sampling-based method followed by comprehensive mapping of all of the reads to accurately determine the abundance of microorganisms. The workflow provides a comprehensive snapshot of the microbial abundance and can easily be used with any state-of-the-art NGS read mapping and assembly algorithms. The list of baseline organisms identified in the normal human gut has clinical applicability as microbiome research moves closer to the bedside. The methods, tools and data from this project can also be used by regulatory scientists to evaluate workflows related to fecal transplant.

In addition to the workflow, this work lays the foundation for an expansive and modular database which can aggregate publicly available data as well as data from contributors to push towards an understanding the baseline human microbiome. This database can serve as a reference in studies of dysbiosis and microbiome associated with diseases. The user-friendly format through FecalBiome report, which contains absolute and relative abundance information about a given sample compared to an average across the entire database allows scientists, clinicians, and eventually patients to understand overview of gut microbiome. This work has the potential to provide a significant impact on regulatory science (e.g., FDA) and standards organization (e.g., NIST) research efforts in this area. For example, GutFeelingKB can potentially allow for rapid assessment of the content of human GI replacement products and, ideally, allow for more expedient review of products. Future studies to advance evidence-based microbiome medicine can be conducted where potential patients identify which outcomes (such as depression, bloating, epilepsy, frequency of common colds, cancer, etc.). For example Apte et al. [20] identified 28 disease-related organisms which can be targeted to evaluate the healthy status of an individual and used to detect disease while FecalBiome report can be used to communicate the microbiome-related health status of an individual between a clinician and patient. Those outcomes will become endpoints in clinical trials or observational studies that demonstrate the effects of various bacteria on the human gut. This type of methodology would tie raw numbers to health states that are meaningful for the general population, ensuring that data gathered are relevant to the patient, and therefore the clinician. This could bring a new, patient-centric perspective to microbiome data use and allow for a greater scope of health data to sit atop metagenomic sequence data. If everyone uses the same set of clinically relevant endpoints, research will be easily comparable across studies and meta-analysis becomes interoperable.

## Supporting information

S1 Fig Quality assurance of one sample. (A) Summary statistics for the read file. (B) ACGT Count: A pie chart displaying the number and percentage of bases present in a read file. (C) Lengthwise Position Count: Displays the number of bases versus position in the read files. (D) Quality Position Count: The average quality score of a position in the reads of a file. (E) Average Quality Per Base: A histogram of the quality score of each base pair. (F) Length Count: A plot of the read length against the number of reads in the sample. (G) Quality Length Count: Shows the average quality score of a read of a given length.
(DOCX)

S2 Fig HIVE-MultiQC output figures. (A) The average quality score for each base shown by sample file. The consistently high-quality score for the forward strand files indicates acceptable sequences for analysis. (B) The relative abundance of each base in each read file. (C) The average quality score for the entire data set, shown by position in the read, is the blue line. The greyed area represents one standard deviation above and below the average.
(DOCX)

S3 Fig Graphical representation of the number of new organisms identified in a sample that is not already present in GutFeelingKB. Each point represents one sample.
(DOCX)

S4 Fig Enterotypes of GW and HMP samples. (DOCX)

S1 Table Anthropomorphic measurements of GW and HMP samples. 1) GW anthropomorphic measurements and the associated value. 2) HMP anthropomorphic measurements and the associated value. 3) Selection criteria.
(XLSX)

S2 Table Nutritional features and the associated values of GW and HMP samples. 1) GW 100 nutritional features and the associated values from NDSR results of GW samples. 2) 100 nutritional features and the associated values of HMP samples.
(XLSX)

S3 Table GW read files information. List of 96 reads file information from 48 GW samples.
(XLSX)

S4 Table GutFeelingKB. Organisms shown in GutFeelingKB are represented by UniProt Proteome IDs. This table lists the same organism’s information through different databases like UniProt, NCBI Assembly, NCBI Taxonomy, NCBI Nucleotide and so on.
(XLSX)

S5 Table Pathogens table. List of well-known gut pathogens that can be misidentified through metagenomics and hence require manual checking.
(XLSX)

S6 Table Blacklist of Filtered-nt. All the removed taxonomy IDs from NCBI-nt to create the Filtered-nt.
(CSV)

S7 Table Proteomes similar to organisms in GutFeelingKB. 157 proteomes IDs in GutFeelingKB were mapped to 128 clusters containing 863 proteomes in Reference Proteomes (RPs) sets (75% cutoff, release_2018_06).
(XLSX)

S8 Table Using the supplementary file (https://doi.org/10.1371/journal.pone.0176555.s003) from Almonacid et al.’s clinical targets paper, we were able to determine that 75 of the organisms listed in GutFeelingKB can be mapped to the targets in that paper. The mapping was done by identifying UniProt Proteome IDs and the RPG cluster that best matched the organism described.
(XLSX)

S9 Table Abundance tables are presented as 7 tables each representing a deferent taxonomy node, including phylum, family, class, order, genus, species, and strain abundance tables. Average abundance, standard deviation, maximal and minimal abundance are provided excluding the organisms with the 0% abundance. The table titled HitList provides the actual number of reads that were mapped.
(XLSX)

S10 Table Associations between clinical metadata and microbial community abundance. (XLSX)

S11 Table Cosine similarity coefficient of correlation. This table demonstrates the range of correlation for features that have been measured, and the organisms that have been detected.
(XLSX)

## References

[1] Huttenhower C, Gevers D, Knight R, Abubucker S, Badger JH, Chinwalla AT, et al Structure, function and diversity of the healthy human microbiome. Nature. 2012;486: 207–214. 10.1038/nature11234 22699609 PMC3564958

[2] Chen X, Ling H-F, Vance D, Shields-Zhou GA, Zhu M, Poulton SW, et al Rise to modern levels of ocean oxygenation coincided with the Cambrian radiation of animals. Nat Commun. 2015;6 10.1038/ncomms8142 25980960 PMC4479002

[3] Wang H, Zheng H, Browne F, Roehe R, Dewhurst RJ, Engel F, et al Integrated metagenomic analysis of the rumen microbiome of cattle reveals key biological mechanisms associated with methane traits. Methods. 2017;124: 108–119. 10.1016/j.ymeth.2017.05.029 28602995

[4] Gao B, Chi L, Mahbub R, Bian X, Tu P, Ru H, et al Multi-Omics Reveals that Lead Exposure Disturbs Gut Microbiome Development, Key Metabolites and Metabolic Pathways. Chem Res Toxicol. 2017;30: 996 10.1021/acs.chemrestox.6b00401 28234468 PMC5654721

[5] Mayer EA, Tillisch K, Gupta A. Gut/brain axis and the microbiota. J Clin Invest. 2015;125: 926–38. 10.1172/JCI76304 25689247 PMC4362231

[6] O’Dwyer DN, Dickson RP, Moore BB. The Lung Microbiome, Immunity, and the Pathogenesis of Chronic Lung Disease. J Immunol. 2016;196: 4839–47. 10.4049/jimmunol.1600279 27260767 PMC4894335

[7] Marshall B, Adams PC. Helicobacter pylori—a Nobel pursuit? Can J Gastroenterol. 2008;22: 895–6. 10.1155/2008/459810 19018331 PMC2661189

[8] Ege MJ. The Hygiene Hypothesis in the Age of the Microbiome. Ann Am Thorac Soc. 2017;14: S348–S353. 10.1513/AnnalsATS.201702-139AW 29161087

[9] Lederberg J, McCray A. ‘Ome Sweet ‘Omics—A Genealogical Treasury of Words | The Scientist Magazine^®^. Sci. 2001;15: 8.

[10] The Integrative Human Microbiome Project: Dynamic Analysis of Microbiome-Host Omics Profiles during Periods of Human Health and Disease. Cell Host Microbe. 2014;16: 276–289. 10.1016/j.chom.2014.08.014 25211071 PMC5109542

[11] NIH HMP Working Group TNHW, Peterson J, Garges S, Giovanni M, McInnes P, Wang L, et al The NIH Human Microbiome Project. Genome Res. 2009;19: 2317–23. 10.1101/gr.096651.109 19819907 PMC2792171

[12] Donia MS, Cimermancic P, Schulze CJ, Wieland Brown LC, Martin J, Mitreva M, et al A systematic analysis of biosynthetic gene clusters in the human microbiome reveals a common family of antibiotics. Cell. 2014;158: 1402–1414. 10.1016/j.cell.2014.08.032 25215495 PMC4164201

[13] Korem T, Zeevi D, Suez J, Weinberger A, Avnit-Sagi T, Pompan-Lotan M, et al Growth dynamics of gut microbiota in health and disease inferred from single metagenomic samples. Science. 2015;349: 1101–1106. 10.1126/science.aac4812 26229116 PMC5087275

[14] Council NR. The New Science of Metagenomics [Internet]. Washington, D.C.: National Academies Press; 2007 10.17226/11902 21678629

[15] Lloyd-Price J, Mahurkar A, Rahnavard G, Crabtree J, Orvis J, Hall AB, et al Strains, functions and dynamics in the expanded Human Microbiome Project. Nature. 2017;550: 61–66. 10.1038/nature23889 28953883 PMC5831082

[16] Proctor LM. The Human Microbiome Project in 2011 and Beyond. Cell Host Microbe. 2011;10: 287–291. 10.1016/j.chom.2011.10.001 22018227

[17] Liang D, Leung RK-K, Guan W, Au WW. Involvement of gut microbiome in human health and disease: brief overview, knowledge gaps and research opportunities. Gut Pathog. 2018;10: 3 10.1186/s13099-018-0230-4 29416567 PMC5785832

[18] Qin J, Li R, Raes J, Arumugam M, Burgdorf KS, Manichanh C, et al A human gut microbial gene catalogue established by metagenomic sequencing. Nature. 2010;464 10.1038/nature08821 20203603 PMC3779803

[19] Stulberg E, Fravel D, Proctor LM, Murray DM, LoTempio J, Chrisey L, et al An assessment of US microbiome research. Nat Microbiol. 2016;1: 15015 10.1038/nmicrobiol.2015.15 27571759

[20] Almonacid DE, Kraal L, Ossandon FJ, Budovskaya YV., Cardenas JP, Bik EM, et al 16S rRNA gene sequencing and healthy reference ranges for 28 clinically relevant microbial taxa from the human gut microbiome. Suchodolski JS, editor. PLoS One. 2017;12: e0176555 10.1371/journal.pone.0176555 28467461 PMC5414997

[21] Nasko DJ, Koren S, Phillippy AM, Treangen TJ. RefSeq database growth influences the accuracy of k-mer-based lowest common ancestor species identification. Genome Biol. 2018;19: 165 10.1186/s13059-018-1554-6 30373669 PMC6206640

[22] NCBI Resource Coordinators NR. Database resources of the National Center for Biotechnology Information. Nucleic Acids Res. 2018;46: D8–D13. 10.1093/nar/gkx1095 29140470 PMC5753372

[23] Shamsaddini A, Pan Y, Johnson WE, Krampis K, Shcheglovitova M, Simonyan V, et al Census-based rapid and accurate metagenome taxonomic profiling. BMC Genomics. 2014;15: 918 10.1186/1471-2164-15-918 25336203 PMC4218995

[24] Lobb B, Kurtz DA, Moreno-Hagelsieb G, Doxey AC. Remote homology and the functions of metagenomic dark matter. Front Genet. 2015;6: 234 10.3389/fgene.2015.00234 26257768 PMC4508852

[25] Bernard G, Pathmanathan JS, Lannes R, Lopez P, Bapteste E. Microbial Dark Matter Investigations: How Microbial Studies Transform Biological Knowledge and Empirically Sketch a Logic of Scientific Discovery. Genome Biol Evol. 2018;10: 707–715. 10.1093/gbe/evy031 29420719 PMC5830969

[26] Scrimshaw NS. INFOODS: the international network of food data systems. Am J Clin Nutr. 1997;65: 1190S–1193S. 10.1093/ajcn/65.4.1190S 9094920

[27] Simonyan V, Mazumder R. High-performance integrated virtual environment (hive) tools and applications for big data analysis. Genes (Basel). 2014;5: 957–981. 10.3390/genes5040957 25271953 PMC4276921

[28] Simonyan V, Chumakov K, Dingerdissen H, Faison W, Goldweber S, Golikov A, et al High-performance integrated virtual environment (HIVE): a robust infrastructure for next-generation sequence data analysis. Database (Oxford). 2016;2016 10.1093/database/baw022 26989153 PMC4795927

[29] Huttenhower C, Fah Sathirapongsasuti J, Segata N, Gevers D, Earl AM, Fitzgerald MG, et al Structure, function and diversity of the healthy human microbiome. Nature. 2012;486: 207–214. 10.1038/nature11234 22699609 PMC3564958

[30] Shamsaddini A, Pan Y, Johnson W, Krampis K, Shcheglovitova M, Simonyan V, et al Census-based rapid and accurate metagenome taxonomic profiling. BMC Genomics. 2014;15: 918 10.1186/1471-2164-15-918 25336203 PMC4218995

[31] Santana-Quintero L, Dingerdissen H, Thierry-Mieg J, Mazumder R, Simonyan V. HIVE-hexagon: High-performance, parallelized sequence alignment for next-generation sequencing data analysis. PLoS One. 2014;9 10.1371/journal.pone.0099033 24918764 PMC4053384

[32] Peng Y, Leung HCM, Yiu SM, Chin FYL. IDBA-UD: a de novo assembler for single-cell and metagenomic sequencing data with highly uneven depth. Bioinformatics. 2012;28: 1420–1428. 10.1093/bioinformatics/bts174 22495754

[33] Altschul SF, Gish W, Miller W, Myers EW, Lipman DJ. Basic local alignment search tool. J Mol Biol. 1990;215: 403–410. 10.1016/S0022-2836(05)80360-2 2231712

[34] Chen C, Natale DA, Finn RD, Huang H, Zhang J, Wu CH, et al Representative Proteomesz: A Stable, Scalable and Unbiased proteome set for sequence analysis and functional annotation. Hoheisel JD, editor. PLoS One. 2011;6: e18910 10.1371/journal.pone.0018910 21556138 PMC3083393

[35] Bateman A, Martin MJ, O’Donovan C, Magrane M, Alpi E, Antunes R, et al UniProt: The universal protein knowledgebase. Nucleic Acids Res. 2017;45: D158–D169. 10.1093/nar/gkw1099 27899622 PMC5210571

[36] Whitman WB, Rainey F, Kämpfer P, Trujillo M, Chun J, DeVos P, et al, editors. Bergey’s Manual of Systematics of Archaea and Bacteria [Internet]. Bergey’s Manual of Systematics of Archaea and Bacteria. Wiley; 2015 10.1002/9781118960608

[37] Santana-Quintero L, Dingerdissen H, Thierry-Mieg J, Mazumder R, Simonyan V. HIVE-hexagon: High-performance, parallelized sequence alignment for next-generation sequencing data analysis. PLoS One. 2014;9: e99033 10.1371/journal.pone.0099033 24918764 PMC4053384

[38] Morgan XC, Tickle TL, Sokol H, Gevers D, Devaney KL, Ward DV, et al Dysfunction of the intestinal microbiome in inflammatory bowel disease and treatment. Genome Biol. 2012;13: R79 10.1186/gb-2012-13-9-r79 23013615 PMC3506950

[39] Okuda S, Tsuchiya Y, Kiriyama C, Itoh M, Morisaki H. Virtual metagenome reconstruction from 16S rRNA gene sequences. Nat Commun. 2012;3: 1203 10.1038/ncomms2203 23149747

[40] Drell T, Larionova A, Voor T, Simm J, Julge K, Heilman K, et al Differences in Gut Microbiota Between Atopic and Healthy Children. Curr Microbiol. 2015;71: 177–183. 10.1007/s00284-015-0815-9 25869237

[41] Schloissnig S, Arumugam M, Sunagawa S, Mitreva M, Tap J, Zhu A, et al Genomic variation landscape of the human gut microbiome. Nature. 2013;493: 45–50. 10.1038/nature11711 23222524 PMC3536929

[42] Zhou W, Gay N, Oh J. ReprDB and panDB: minimalist databases with maximal microbial representation. Microbiome. 2018;6: 15 10.1186/s40168-018-0399-2 29347966 PMC5774170

[43] Qin J, Li R, Raes J, Arumugam M, Burgdorf KS, Manichanh C, et al A human gut microbial gene catalogue established by metagenomic sequencing. Nature. 2010;464: 59–65. 10.1038/nature08821 20203603 PMC3779803

[44] Li J, Jia H, Cai X, Zhong H, Feng Q, Sunagawa S, et al An integrated catalog of reference genes in the human gut microbiome. Nat Biotechnol. 2014;32: 834–841. 10.1038/nbt.2942 24997786

[45] Yatsunenko T, Rey FE, Manary MJ, Trehan I, Dominguez-Bello MG, Contreras M, et al Human gut microbiome viewed across age and geography. Nature. 2012;486: 222–227. 10.1038/nature11053 22699611 PMC3376388

[46] Nishijima S, Suda W, Oshima K, Kim S-W, Hirose Y, Morita H, et al The gut microbiome of healthy Japanese and its microbial and functional uniqueness. DNA Res. 2016;23: 125–133. 10.1093/dnares/dsw002 26951067 PMC4833420

[47] Qin N, Yang F, Li A, Prifti E, Chen Y, Shao L, et al Alterations of the human gut microbiome in liver cirrhosis. Nature. 2014;513: 59–64. 10.1038/nature13568 25079328

[48] Gal-Mor O, Boyle EC, Grassl GA. Same species, different diseases: How and why typhoidal and non-typhoidal Salmonella enterica serovars differ [Internet]. Frontiers in Microbiology. Frontiers; 2014 p. 391 10.3389/fmicb.2014.00391 25136336 PMC4120697

[49] Strauss J, Kaplan GG, Beck PL, Rioux K, Panaccione R, DeVinney R, et al Invasive potential of gut mucosa-derived fusobacterium nucleatum positively correlates with IBD status of the host. Inflamm Bowel Dis. 2011;17: 1971–1978. 10.1002/ibd.21606 21830275

[50] Bourne PE, Bonazzi V, Dunn M, Green ED, Guyer M, Komatsoulis G, et al The NIH Big Data to Knowledge (BD2K) initiative. J Am Med Inform Assoc. 2015;22: 1114 10.1093/jamia/ocv136 26555016 PMC5009910

[51] Arumugam M, Raes J, Pelletier E, Le Paslier D, Yamada T, Mende DR, et al Enterotypes of the human gut microbiome. Nature. 2011;473: 174–180. 10.1038/nature09944 21508958 PMC3728647

[52] Wexler HM. Bacteroides: the good, the bad, and the nitty-gritty. Clin Microbiol Rev. 2007;20: 593–621. 10.1128/CMR.00008-07 17934076 PMC2176045

[53] Mazmanian SK, Round JL, Kasper DL. A microbial symbiosis factor prevents intestinal inflammatory disease. Nature. 2008;453: 620–625. 10.1038/nature07008 18509436

[54] Coyne MJ, Comstock LE. Niche-Specific Features of the Intestinal Bacteroidales. J Bacteriol. 2008;190: 736 10.1128/JB.01559-07 17993536 PMC2223690

[55] O’Callaghan A, van Sinderen D. Bifidobacteria and Their Role as Members of the Human Gut Microbiota. Front Microbiol. 2016;7: 925 10.3389/fmicb.2016.00925 27379055 PMC4908950

[56] Xiao M, Xu P, Zhao J, Wang Z, Zuo F, Zhang J, et al Oxidative stress-related responses of Bifidobacterium longum subsp. longum BBMN68 at the proteomic level after exposure to oxygen. Microbiology. 2011;157: 1573–1588. 10.1099/mic.0.044297-0 21349974

[57] Fukuda S, Toh H, Hase K, Oshima K, Nakanishi Y, Yoshimura K, et al Bifidobacteria can protect from enteropathogenic infection through production of acetate. Nature. 2011;469: 543–547. 10.1038/nature09646 21270894

[58] Hao Y, Huang D, Guo H, Xiao M, An H, Zhao L, et al Complete genome sequence of bifidobacterium longum subsp. longum BBMN68, a new strain from a healthy Chinese centenarian. J Bacteriol. 2011;193: 787–788. 10.1128/JB.01213-10 21097614 PMC3021241

[59] Kim N, Kunisawa J, Kweon M-N, Eog Ji G, Kiyono H. Oral feeding of Bifidobacterium bifidum (BGN4) prevents CD4+ CD45RBhigh T cell-mediated inflammatory bowel disease by inhibition of disordered T cell activation. Clin Immunol. 2007;123: 30–39. 10.1016/j.clim.2006.11.005 17218154

[60] Shelburne SA, Sahasrabhojane P, Saldana M, Yao H, Su X, Horstmann N, et al Streptococcus mitis strains causing severe clinical disease in cancer patients. Emerg Infect Dis. 2014;20: 762–771. 10.3201/eid2005.130953 24750901 PMC4012796

[61] Palmer C, Bik EM, DiGiulio DB, Relman DA, Brown PO. Development of the Human Infant Intestinal Microbiota. Ruan Y, editor. PLoS Biol. 2007;5: e177 10.1371/journal.pbio.0050177 17594176 PMC1896187

[62] Miquel S, Peyretaillade E, Claret L, de Vallée A, Dossat C, Vacherie B, et al Complete Genome Sequence of Crohn’s Disease-Associated Adherent-Invasive E. coli Strain LF82. Ahmed N, editor. PLoS One. 2010;5: e12714 10.1371/journal.pone.0012714 20862302 PMC2941450

[63] Feng Q, Liang S, Jia H, Stadlmayr A, Tang L, Lan Z, et al Gut microbiome development along the colorectal adenoma–carcinoma sequence. Nat Commun. 2015;6: 6528 10.1038/ncomms7528 25758642

[64] Howe A, Chain PSG. Challenges and opportunities in understanding microbial communities with metagenome assembly (accompanied by IPython Notebook tutorial). Front Microbiol. 2015;6: 678 10.3389/fmicb.2015.00678 26217314 PMC4496567

[65] Land M, Hauser L, Jun S-R, Nookaew I, Leuze MR, Ahn T-H, et al Insights from 20 years of bacterial genome sequencing. Funct Integr Genomics. 2015;15: 141–61. 10.1007/s10142-015-0433-4 25722247 PMC4361730

[66] Zackular JP, Rogers MaM, Ruffin MT, Schloss PD. The Human Gut Microbiome as a Screening Tool for Colorectal Cancer. Cancer Prev Res. 2014;7: 1940–6207. CAPR-14-0129-. 10.1158/1940-6207.CAPR-14-0129 25104642 PMC4221363

[67] Silverman E, Niehaus A. NHGRI Genomic Medicine IX: NHGRI’s Genomic Medicine Portfolio–Bedside to Bench. https://www.genome.gov/Multimedia/Slides/GM9/gm9_summary_final_GM9full_SilvermanE.pdf

[68] Donaldson GP, Lee SM, Mazmanian SK. Gut biogeography of the bacterial microbiota. Nat Rev Microbiol. 2016;14: 20–32. 10.1038/nrmicro3552 26499895 PMC4837114

[69] Dao MC, Everard A, Aron-Wisnewsky J, Sokolovska N, Prifti E, Verger EO, et al Akkermansia muciniphila and improved metabolic health during a dietary intervention in obesity: relationship with gut microbiome richness and ecology. Gut. 2016;65: 426–36. 10.1136/gutjnl-2014-308778 26100928

[70] Bloom SM, Bijanki VN, Nava GM, Sun L, Malvin NP, Donermeyer DL, et al Commensal Bacteroides species induce colitis in host-genotype-specific fashion in a mouse model of inflammatory bowel disease. Cell Host Microbe. 2011;9: 390–403. 10.1016/j.chom.2011.04.009 21575910 PMC3241010

[71] Rubinstien EM, Klevjer-Anderson P, Smith CA, Drouin MT, Patterson JE. Enterobacter taylorae, a new opportunistic pathogen: report of four cases. J Clin Microbiol. 1993;31: 249–54. Available: http://www.ncbi.nlm.nih.gov/pubmed/8381808 8381808 10.1128/jcm.31.2.249-254.1993 PMC262744

[72] Flint HJ, Scott KP, Duncan SH, Louis P, Forano E. Microbial degradation of complex carbohydrates in the gut. Gut Microbes. 2012;3: 289–306. 10.4161/gmic.19897 22572875 PMC3463488

