# Supplementary material for: Baseline human gut microbiota profile in healthy people and standard reporting template
Source: PLoS One. 2019 Sep 11;14(9):e0206484. doi: 10.1371/journal.pone.0206484 (PMC6738582; doi:10.1371/journal.pone.0206484)
Supplement: S1 Fig — (A) Summary statistics for the read file. (B) ACGT Count: A pie chart displaying the number and percentage of bases present in a read file. (C) Lengthwise Position Count: Displays the number of bases versus position in the read files. (D) Quality Position Count: The average quality score of a position in the reads of a file. (E) Average Quality Per Base: A histogram of the quality score of each base pair. (F) Length Count: A plot of the read length against the number of reads in the sample. (G) Quality Length Count: Shows the average quality score of a read of a given length. (DOCX) [file pone.0206484.s001.docx]

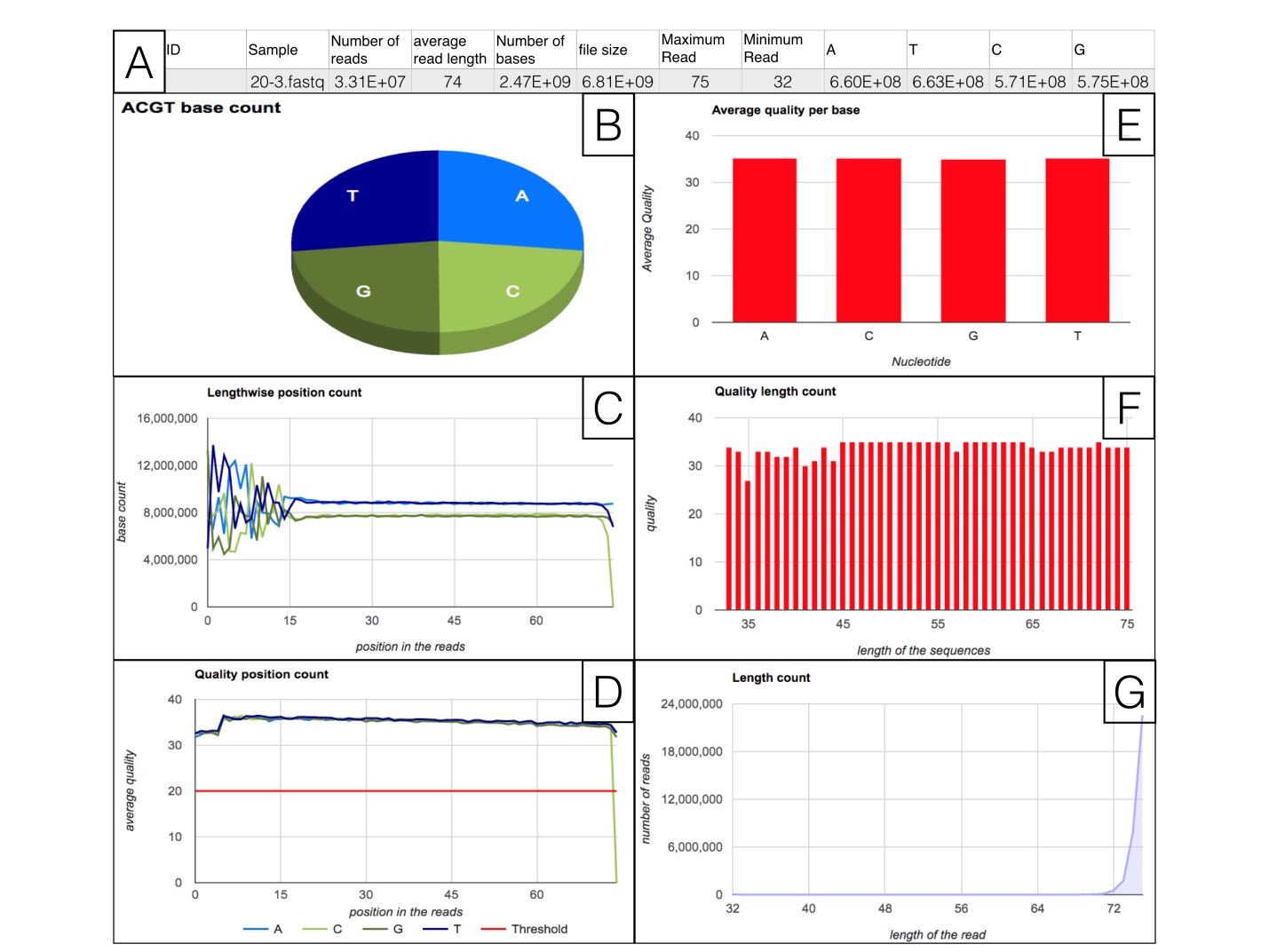


S1 Fig. Quality assurance of one sample. (A) Summary statistics for the read file. (B) *ACGT Count:* A pie chart displaying the number and percentage of bases present in a read file. (C) *Lengthwise Position Count*: Displays the number of bases versus position in the read files. (D) *Quality Position Count:* The average quality score of a position in the reads of a file. (E) *Average Quality Per Base:* A histogram of the quality score of each base pair. (F) *Length Count:* A plot of the read length against the number of reads in the sample. (G) *Quality Length Count:* Shows the average quality score of a read of a given length.
