# Supplementary material for: Baseline human gut microbiota profile in healthy people and standard reporting template
Source: PLoS One. 2019 Sep 11;14(9):e0206484. doi: 10.1371/journal.pone.0206484 (PMC6738582; doi:10.1371/journal.pone.0206484)
Supplement: S2 Fig — (A) The average quality score for each base shown by sample file. The consistently high-quality score for the forward strand files indicates acceptable sequences for analysis. (B) The relative abundance of each base in each read file. (C) The average quality score for the entire data set, shown by position in the read, is the blue line. The greyed area represents one standard deviation above and below the average. (DOCX) [file pone.0206484.s002.docx]

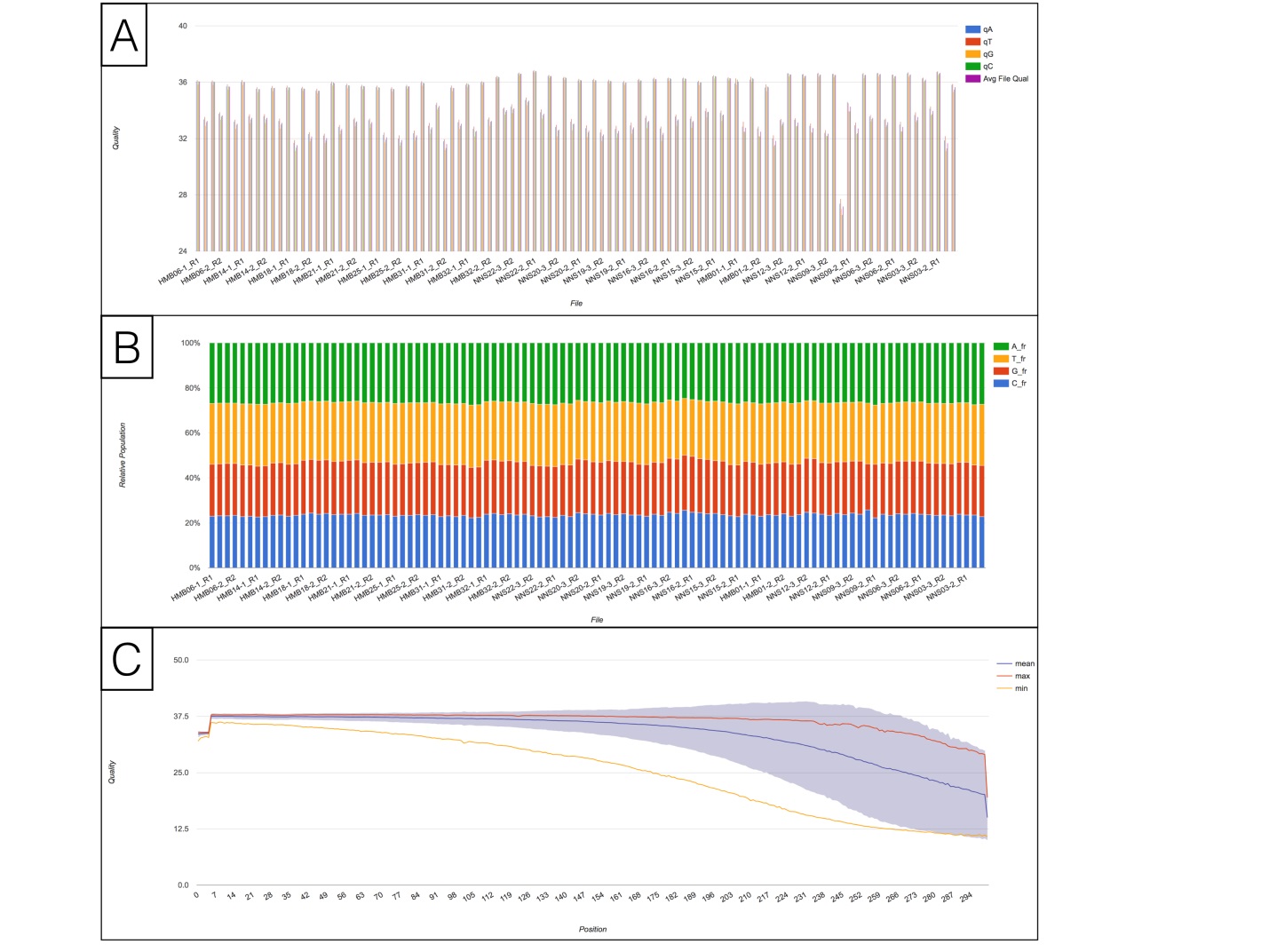


S2 Fig. HIVE-MultiQC output figures. (A) The average quality score for each base shown by sample file. The consistently high-quality score for the forward strand files indicates acceptable sequences for analysis. (B) The relative abundance of each base in each read file. (C) The average quality score for the entire data set, shown by position in the read, is the blue line. The greyed area represents one standard deviation above and below the average.
