# Supplementary material for: Baseline human gut microbiota profile in healthy people and standard reporting template
Source: PLoS One. 2019 Sep 11;14(9):e0206484. doi: 10.1371/journal.pone.0206484 (PMC6738582; doi:10.1371/journal.pone.0206484)
Supplement: S3 Fig — Each point represents one sample. (DOCX) [file pone.0206484.s003.docx]

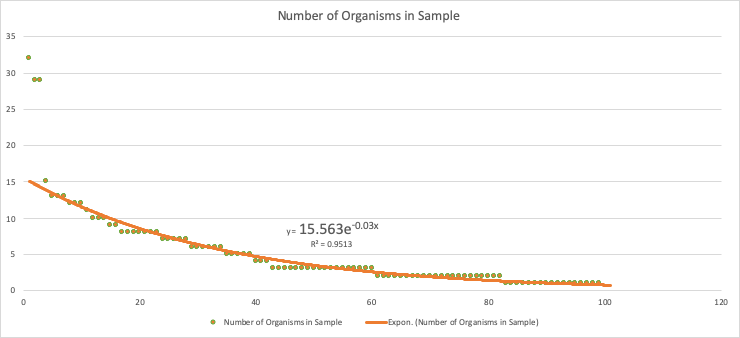


S3 Fig. Graphical representation for the number of organisms in identified in a sample. Each point is the value for one sample. The line is an exponential trendline.
