# Supplementary figures and images for: Baseline human gut microbiota profile in healthy people and standard reporting template
Source: PLoS One. 2019 Sep 11;14(9):e0206484. doi: 10.1371/journal.pone.0206484 (PMC6738582; doi:10.1371/journal.pone.0206484)

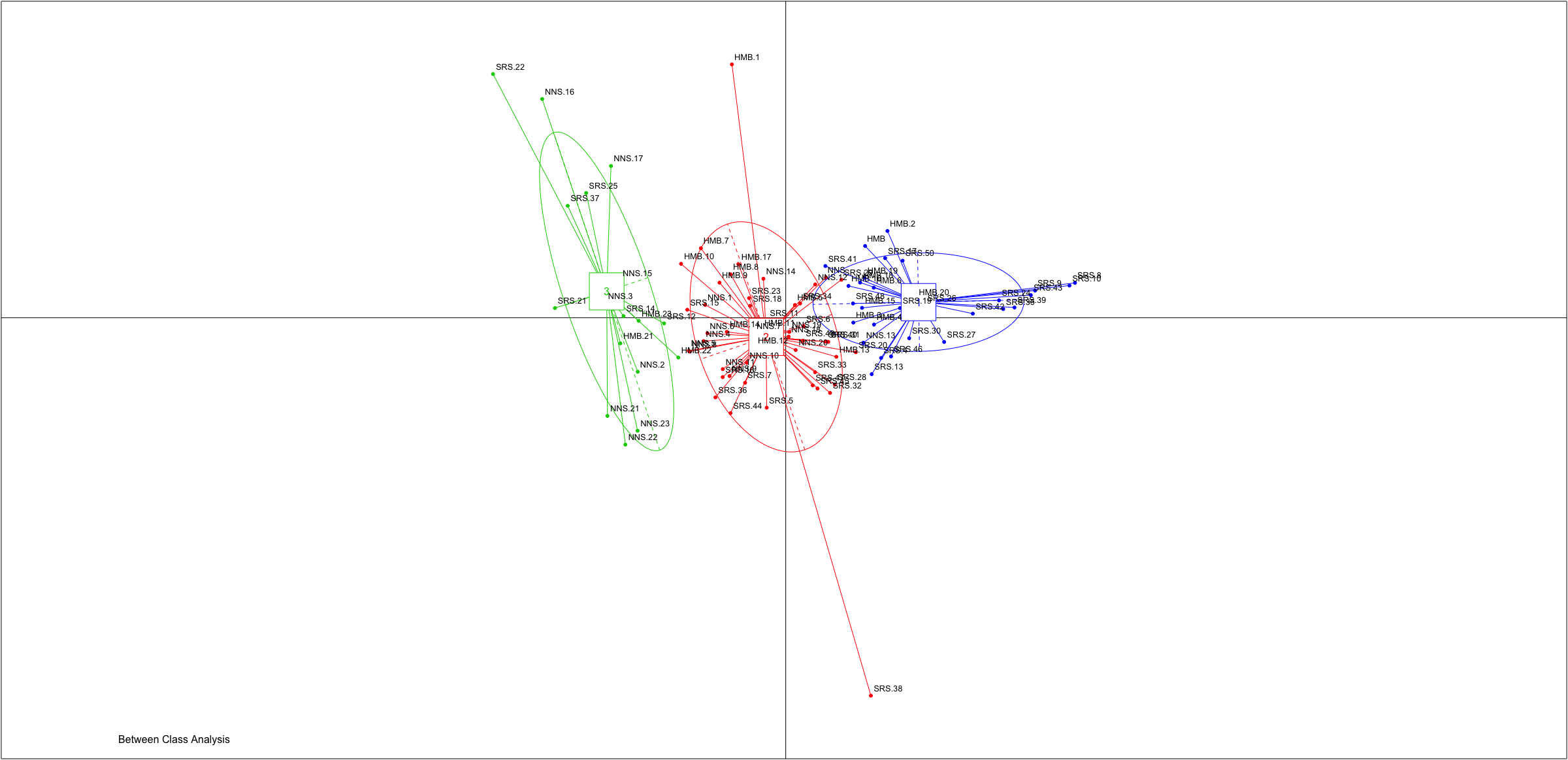


S4 Fig. Enterotypes of GW and HMP samples.

Supplement: S4 Fig — (DOCX) [file pone.0206484.s004.docx]
